# Effect of a harmonic surface pressure on wave propagation over a beach

**DOI:** 10.1038/s41598-024-67443-6

**Published:** 2024-07-30

**Authors:** N. S. Abdelrahman, M. S. Abou-Dina, A. F. Ghaleb

**Affiliations:** 1 Department of Mathematics, Faculty of Science, Suez Canal University, Ismailia, Egypt; 2 Department of Mathematics, Faculty of Science, Cairo University, Giza, 12613 Egypt

**Keywords:** Harmonic wave propagation, Potential flow, Surface pressure, Linear theory, Finite Fourier transform, Energy transfer, Water power conversion, Engineering, Mathematics and computing, Physics

## Abstract

The objective is to study the harmonic forced wave motion over a beach by a finite Fourier transform technique. The constructed approximate solution has a logarithmic singularity at the shoreline. It accounts for reflexion and local perturbations. Trapping of waves may take place for particular choices of the applied surface pressure excess. The case of a wave incident against a cliff with horizontal bottom is solved exactly. The method deals invariably with a variety of bottom shapes, including the case where there is an additional corrugation of the bottom on a finite interval. Other bottom boundary conditions than impermeability can be treated as well. The results may be of interest in several practical applications, in particular the evaluation of the reflected wave. Numerical applications for a plane sloping beach, a parabolic-type beach and a shelf-type beach are presented and the systems of streamlines have been drawn over and in the proximity of the beach.

## Introduction

The investigation of wave motion on beaches may be traced back to the end of the nineteenth century. The dissipation phenomena during wave propagation over beaches have been extensively studied. On the other hand, the evaluation of wave reflection by the beach is still awaiting further investigation. The research work was mainly undertaken using the techniques of complex analysis, involving a great deal of complexity of the calculations. One of the early contributions to the subject is due Lewy^1^ who studies progressive water waves on beaches of constant slope for special values of the angle of inclination. Friedrichs^2^ presents an asymptotic representation of the solution for small slopes. John^3^ considers a barrier inclined at a special angle in water of infinite depth. Isaacson^4^ investigates progressing gravity waves over a plane sloping bottom. Peters^5^ studies the effect of a surface mat on water waves. He considers water wave propagation over a beach under a different bottom condition. The problem formulation and solution for waves on beaches may be found in Stoker^6^, Ch. 5, with a discussion on the validity of the solutions under different theories. Wehausen and Laitone^7^, p.537 give a detailed description of the problem under the general title of plane wave motion in unbounded regions with fixed boundaries. Lehman and Lewy^8^ discuss the uniqueness problem for water waves on sloping beaches and the boundedness of solutions. Peregrine^9^ studies the propagation of long waves in water of variable depth through nonlinear equations. Experimental studies on wave reflection by a sloping beach in a tank and the dependence of the reflection coefficient on wave steepness were carried out by Taira and Nagata^10^. Tuck and Hwang^11^ investigate the linear propagation of long waves on a uniformly sloping beach. Near-shore large amplitude waves are also investigated using the nonlinear theory. Suhayda^12^ presents measurements associated with standing waves beaches. Sachdev and Seshadri^13^ propose an approximate analytical solution to the problem of motion of a bore on a sloping beach. Svendsen and Hansen^14^ investigate two-dimensional time-periodic water waves on a gently sloping bottom in the long-wave limit. They derive solutions up to the second-order degree of smallness. Mahony and Pritchard^15^ study wave reflexion from beaches and the dependence on friction at the bottom of the reflexion coefficient. Peregrine^16^ presents an overview of wave breaking on beaches. Ehrenmark^17^ considers the problem of a train of infinitesimal waves propagating over a uniformly sloping beach and discusses solutions having singularities of different orders at the shoreline. Miles^18^ studies wave reflection from a gently sloping beach within the linear theory. Mandal and Kundu^19^ re-investigate the two-dimensional problem of incoming wave against a cliff by Fourier transform. They present a simplified solution which includes a logarithmic singularity at the shoreline. The effect of surface tension is considered. Chakrabarti^20^ studies the propagation of waves against a cliff under the assumptions of linearized theory. His solution exhibits a source/sink type behavior of the velocity potential at the shore-line. Gupta^21^ proposes an analytic solution describing the motion of a bore over a uniformly sloping beach for the supercritical case. McIver^22^ provides an example of non-uniqueness in the twodimensional linear water wave problem. Javam et al.^23^ undertake a numerical study of internal wave reflection from sloping boundaries within a nonlinear theory. Ehrenmark^24^ studies wave trapping above a plane beach by partially or totally submerged obstacles within the linear theory. He underlines a case of non-uniqueness for the water wave problem on a beach. Liu et al.^25^ obtain analytical solutions for forced long waves on a sloping beach. Comparison is carried out with previous numerical solutions. Dias and Dutych^26^, Fujima^27^ and Helal^28^ study tsunami modeling and runup on beaches. Bukreev^29^ presents experimental results concerning the reflection of a nonlinear wave from a vertical wall.

Simarro et al.^30^ present a fully nonlinear Boussinessq-type model with several free parameters for the study of water waves in fluids of varying depth. Martin and Taskinen and Martin et al.^31,32^ study linear wave propagation in a pond with shallow beach. They show that the problem may have some continuous spectrum, in spite of the boundedness of the solution domain. Xu and Dias^33^ take a look back at old water wave solutions on a uniformly sloping beach and present four different standing wave solutions to this problem. Gallerano et al.^34,35^ present numerical simulation and shock capturing models for free surface flow and runup over beaches based on a nonlinear model. Durán et al.^36^ proposed a modification of the governing equations of long-wave propagation in shallow water that include well-conditioned dispersive terms to achieve efficient and stable run-up computations in the swash zone.

Wave propagation in shallow water over topography, and tsunami runup over beaches were studied theoretically and numerically by many authors, among whom Dobrokhotov and Nazaikinskii^37^, Bihlo and Popovych^38^, Zhang et al.^39^, Zhu and Wang^40^.

The existing literature deals mainly with uniformly sloping beaches with extension to deep water, or with vertical barriers and cliffs in water of finite or infinite depth. More investigations are still needed to deal with general beach shapes. The overwhelming majority of work does not include numerics.

The present work investigates approximate singular solutions to the problem of harmonic wave propagation over a beach in the presence of a pressure excess applied to a finite portion of the water surface. The solution has a logarithmic singularity at the shoreline, and is otherwise smooth inside the flow domain. In view of the flow obstruction by the beach, the logarithmic singularity is necessary as it provides a harmonic source/sink that takes part in the balance of mass. Explicit formulae are obtained for the strength of the logarithmic source, the reflexion coefficient and the coefficients of local perturbations in terms of unknown coefficients. These latter are calculated approximately from the satisfaction of the impermeability condition on the bottom. Cases are noted where wave trapping takes place for certain distributions of the surface pressure excess. The case of an incident wave against a cliff is solved exactly. The method may be used to treat a variety of bottom shapes, as well as cases when the bottom has an additional corrugation on a finite interval. Other boundary conditions (cf.^5^) than impermeability may be treated by the same method equally well. Computations have been carried out for a plane sloping beach, a parabolic-type beach and a shelf-type beach. The systems of streamlines have been drawn over the beach and over the adjacent part with horizontal bottom. The obtained results clearly indicate that there is complete reflection of the incoming harmonic wave, which means that a system of standing waves establishes in the channel. It is believed that this stems from the particular choice of a length parameter included in Fourier finite transform. The action of the excess surface pressure is to alter the shape of the streamlines and the values of the streamfunction along the different streamlines. The present method performs well in the interval $1 \le \kappa \le 2$. For larger values of $\kappa$, the errors in satisfying the different boundary conditions start to increase. Work is in progress to discover other types of solutions.

## Problem formulation and frame of reference

**Figure 1:**
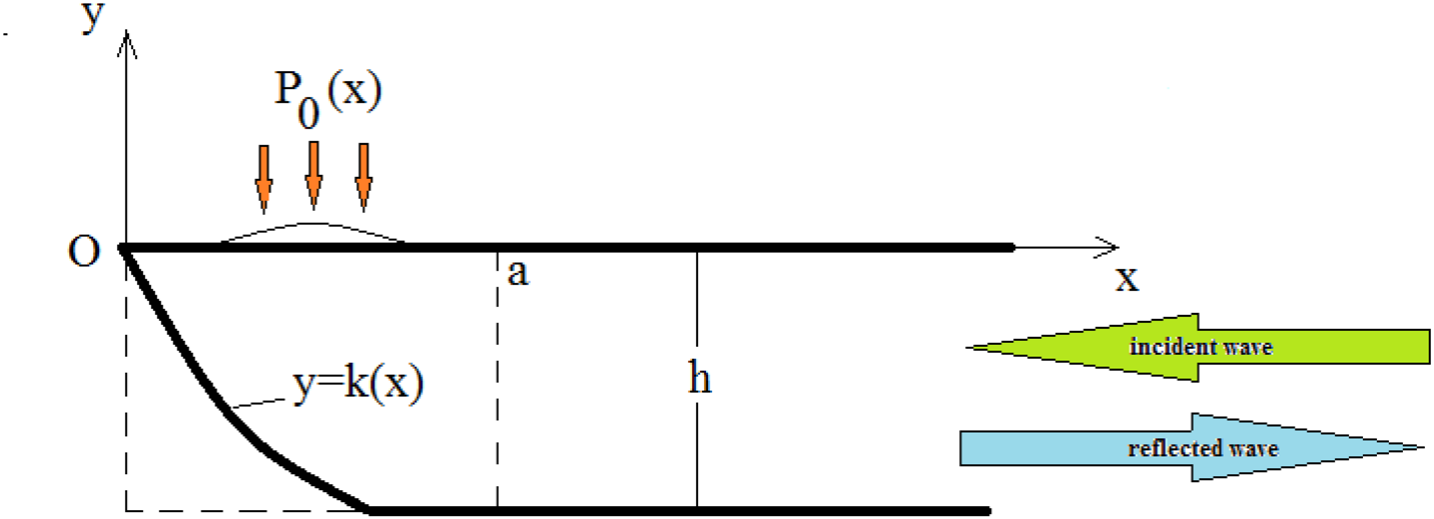
Solution regions for a beach.

Consider the incompressible, two-dimensional, potential flow of an inviscid fluid of constant density ($\rho = 1$) in a semi-infinite channel with finite end in the form of a sloping beach, and a constant depth otherwise. The fluid motion is generated by an incident wave of amplitude $I_0$ and harmonic time dependence of frequency $\omega$, in addition to a given external pressure excess of the same harmonic time dependence, applied on a finite portion of the water surface. A system of outgoing waves establishes in the fluid body. It is required to determine the resulting fluid motion.

A rectangular frame of reference is used to describe the motion, with origin *O* and *x*-axis along the mean level of the water surface in the direction of propagation of the incident wave, and *y*-axis vertically upwards, as illustrated in Fig. 1. Since the incident wave and the surface pressure excess have the same harmonic time dependence, it is reasonable to assume that the velocity potential function will share the same time dependence. Thus, the time dependent velocity potential $\phi (x,y,t)$ may be written as:

1 $$\begin{aligned} \phi (x,y,t)=Re{\left[ \phi (x,y)e^{-i\omega t}\right] } , \end{aligned}$$

where $\phi (x,y)$ is a complex function of *x* and *y*. Time can now be eliminated from all governing equations and boundary conditions.

The equation of the bottom is of the form $y=k(x)$, where

2 $$\begin{aligned} k(x)= {\left\{ \begin{array}{ll} f(x) , \quad 0 \le x \le b,\\ -h, \quad x \ge b, \end{array}\right. } \end{aligned}$$

with

3 $$\begin{aligned} f(0)=0, \qquad f(b) = -h. \end{aligned}$$

The governing equations of the linearized theory of motion are best obtained from the nonlinear equations by expansion in a small parameter. The linear approximation imposes restrictions on the value of the dimensionless parameter $\kappa$ defined below and on the free surface elevation $\eta$ above a mean horizontal line. This elevation is of the same order of magnitude as the small parameter $\varepsilon$ used in the linearization process. According to^41^, the domain of applicability of the linear theory of motion roughly corresponds to

$$\begin{aligned} 0.08< \kappa , \quad \varepsilon \kappa < 0.04. \end{aligned}$$

The reader is kindly referred to^41,42^ for details:

(i) In the fluid mass $\left( -k(x)\le y\le 0, \quad f^{-1}(y) \le x< \infty \right)$, the condition of incompressibility yields:
  4 $$\begin{aligned} \frac{\partial ^{2}\phi }{\partial x^{2}}+\frac{\partial ^{2}\phi }{\partial y^{2}}=0. \end{aligned}$$
(ii) At the upper bound of the domain of the problem $\left( y=0, \quad x \ge 0\right)$: An excess oscillatory external pressure of finite support is applied, having the same order of magnitude and the same harmonic time dependence as those of the incident wave. In the absence of such excess pressure, one usually assumes a constant pressure at the surface, this may be taken equal to zero. If the external oscillatory excess pressure has the form $P_{ex}(x,t)=P_0(x) e^{-i \omega t}$, the boundary condition at $y=0$ in the frame of the linearized theory which expresses the impermeability of the upper fluid surface, is written as:
  5 $$\begin{aligned} \frac{\partial \phi }{\partial y}-\frac{\omega ^{2}}{g}\phi = \frac{i \omega }{\rho g} P_0(x), \end{aligned}$$
  where *g* is the acceleration of gravity. Function $P_0(x)$ is assumed to have a finite support $\{x: \alpha \le x \le \beta \}$, and is continuous on it, except for a finite number of finite discontinuities. Assuming continuity of the time-independent surface elevation $\eta (x)$ of the upper fluid surface above a horizontal equilibrium level ($y=0$), a jump in the pressure function $P_0(x)$ at a certain point induces a similar behavior of the velocity potential $\phi$ at the same point.
(iii) On the bottom of the channel $\left( y=k(x),\,0 \le x<\infty \right)$ and on the surfaces of the obtacles, the normal velocity component vanishes:
  6 $$\begin{aligned} \frac{\partial \phi }{\partial n}=0. \end{aligned}$$
(iv) The radiation condition at infinity $\left( x \rightarrow \infty \right)$ stating that: no wave is coming from infinity except the prescribed incident one.

The time-dependent water surface elevation

7 $$\begin{aligned} \eta ^{*}(x,\,t)=Re{\left[ \eta (x)e^{-i\omega t}\right] } , \end{aligned}$$

and the time-dependent pressure at any point inside the fluid as the sum of the usual hydrostatic pressure and the surface excess pressure:

8 $$\begin{aligned} P^{*}(x,y,t)=-\rho gy+Re{\left[ P(x,y)e^{-i\omega t}\right] } , \end{aligned}$$

are expressed in terms of $\eta (x)$ and *P*(*x*, *y*):

9 $$\begin{aligned} \eta (x)&= \frac{i\omega }{ g}\phi (x,0) - \frac{1 }{\rho g} P_0(x), \end{aligned}$$

10 $$\begin{aligned} P\left( x,\,y\right)&=i\rho \omega \, \phi (x,y) + P_0(x). \end{aligned}$$

hence finally in terms of the time independent velocity potential $\phi (x,y)$.

We do an analytic continuation of the harmonic function $\phi$ to the whole rectangle $V = \left( 0\,\le x\le a,\,-h\,\le y\le 0\right)$. Without ambiguity, the same notation $\phi (x,y)$ will be retained for the extended function.

## Method of solution

The flow domain is divided into two regions separated by a vertical line as in Fig. 1: A volume $V^0$ bounded by the *x*-axis, the vertical line $x=a$ and the bottom line, and an unbounded volume $V^+$ to the right of $V^0$. Constant *a* is chosen so that the finite support $(\alpha , \beta )$ of the pressure excess satisfies $(0< \alpha< \beta <a)$, and the bottom line is horizontal for $x \ge a$. The solution in the semi-infinite region is obtained by separation of variables and consists of an outgoing wave and local perturbations, in addition to an incident wave. Details of the solution may be found in^41^:

1. Denote by $\lambda _{0}$ the only real positive root of the transcendental equation
  $$\begin{aligned} \lambda h \,\tanh \lambda h=\frac{\omega ^{2} h}{g} = \kappa \end{aligned}$$
  corresponding to the Sturm–Liouville eigenvalue problem
  $$\begin{aligned} - \frac{d^2 F_1}{dy^2} = - \lambda ^2 \, F_1, \, \, \frac{d F_1}{d y} - \frac{\omega ^2}{g} \phi =0 \, \, \text {at y=0}, \, \frac{d F_1}{d y} = 0 \, \text {at y = -h}. \end{aligned}$$
2. Denote by $\lambda _{p}$ $\left( p=1,2,3,...\right)$ the positive roots of the transcendental equation
  $$\begin{aligned} \lambda h \,\tan \lambda h=-\kappa , \end{aligned}$$
  arranged by increasing magnitude. This arises from the Sturm-Liouville eigenvalue problem:
  $$\begin{aligned} - \frac{d^2 F_2}{dy^2} = \lambda ^2 \, F_2, \, \, \frac{d F_2}{d y} - \frac{\omega ^2}{g} \phi =0 \, \, \text {at y=0}, \, \frac{d F_2}{d y} = 0 \, \text {at y = -h} \end{aligned}$$

Related to these Sturm–Liouville problems is the following complete and orthogonal set of functions $\{ \cosh \lambda _0 (y+h), \cos \lambda _p (y+h), p=1,2, \cdots \}$ on the interval $[-h,0]$.

The solution for the total time independent velocity potential in the region $V^+$ satisfying the boundedness requirement at infinity is taken as:

11 $$\begin{aligned} \Phi ^{+}(x,y)= & {} \left\{ I_{0}\,e^{-i\lambda _{0}x}+R_{0}\,e^{i\lambda _{0}x}\right\} \cosh \lambda _{0}(y+h) + \sum _{p=1}^{\infty }R_{p} \cos \lambda _{p}(y+h)\,e^{-\lambda _{p} (x-a)} . \end{aligned}$$

The quantities $R_p$ represent the so-called local perturbation coefficients, necessary to achieve continuity of the flow in the vicinity of the separation vertical line at $x= a$.

On the right bound of the domain $V^0\,\,\left( x=a,\,-h\,\le y\le 0\right)$, the continuity of the pressure and the velocity yields the following expressions for the solution $\Phi ^0(x,y)$:

12 $$\begin{aligned} \Phi ^0(a,y)= & {} \left( I_{0} e^{-i\lambda _{0}a}+R_{0} e^{i\lambda _{0}a}\right) \cosh \lambda _{0}(y+h) + \sum _{p=1}^{\infty }R_{p} \cos \lambda _{p}(y+h), \end{aligned}$$

13 $$\begin{aligned} \frac{\partial \Phi ^0}{\partial x}(a,y)= & {} i\lambda _{0} \left( - I_{0} e^{-i\lambda _{0}a}+R_{0} e^{i\lambda _{0}a}\right) \cosh \lambda _{0}(y+h) - \sum _{p=1}^{\infty }\lambda _{p}R_{p} \cos \lambda _{p}(y+h). \end{aligned}$$

Introduce the finite cosine Fourier transform of the surface pressure excess function $P_0(x)$

14 $$\begin{aligned} \rho g h a P_{m}= \int _{0}^{a}\,P_0(x)\,\cos \frac{m \pi x }{a} \,dx,\,m=0,1,2, \ldots \end{aligned}$$

The inversion formula reads

15 $$\begin{aligned} P_0(x)=\sum _{m=0}^{\infty }q_m^0 \, \left( \rho g h P_{m} \right) \cos \frac{m \pi x}{a}, \end{aligned}$$

where $q_m^0 = 2 - \delta _m^0$, $\delta _{m}^{0}$ is the Krönecker delta symbol. Under the above assumptions, coefficients $P_m$ tend to zero at least like $\displaystyle \frac{1}{m}$ as *m* grows indefinitely large.

To write an expression for the solution in the bounded region $V^0$, we refer to a remark by Stoker^6^, p. 81 : “... we expect to find two solutions of our problem which behave differently at the origin and at infinity. At the origin, in particular, we expect to find one solution to be bounded and the other to have a logarithmic singularity”  and another one by Wehausen and Laitone^7^, p. 537: “... All these authors restricted the solution to be bounded everywhere. This has the effect of excluding a physically important class of solutions with singularities at the origin”. Following these remarks, we consider a velocity potential function that belongs to a functional space whose functions possess a logarithmic singularity at the origin. A convenient representation of the solution for the velocity potential in the region $V^0$ is taken as:

16 $$\begin{aligned} \Phi ^{0}(x,y) = \Phi (x,y) + E \, \cosh \frac{2N \pi y}{a} \cos \frac{2N \pi x}{a} + C \, Z(x,y), \end{aligned}$$

where *E* and *C* are unknown coefficients to be determined in the process of the solution, *N* is a positive integer the value of which will be fixed later on, $\Phi (x,y)$ is regular in the extended domain *V*, and

17 $$\begin{aligned} Z(x,y)= & {} \frac{1}{4} \left[ \ln \frac{\sqrt{x^2+y^2}}{h} + \ln \frac{\sqrt{x^2+(y+2h)^2}}{h} \right. \nonumber \\- & {} \left. \ln \frac{\sqrt{(x-2a)^2+y^2}}{h} - \ln \frac{\sqrt{(x-2a)^2+(y+2h)^2}}{h} \right] . \end{aligned}$$

The extracted term from $\Phi$ with multiplicative constant *E* is necessary when treating the special case of a cliff with horizontal bottom. As to the function *Z*(*x*, *y*), it has a logarithmic singularity at the origin of coordinates through its first component, while the other three components are regular functions in the flow domain and on its boundary. This function has been chosen so as to make the calculations simpler due to the following relations:

18 $$\begin{aligned} Z(a,y) = 0, \quad \left. \frac{\partial Z}{\partial y}\right| _{x=a} = 0, \quad \left. \frac{\partial }{\partial y} \left( \frac{\partial Z}{\partial x} \right) \right| _{\begin{array}{c} x=a \\ \, \, \, \, y=-h \end{array}} = 0. \end{aligned}$$

The presence of the singularity at the shoreline with the beach should not cause concern, because a whole interval at this point lies outside the region of applicability of the linear theory due to wave breaking (cf.^15^, p. 810).

The next step is to expand the horizontal derivative of the extended harmonic function $\Phi$ on the newly introduced boundary at $x=0$ in terms of the above-mentioned complete orthogonal family of functions as:

19 $$\begin{aligned} \frac{\partial \Phi }{\partial x}(0,y) = i\lambda _{0} B_0 \cosh \lambda _{0}(y+h) + \sum _{p=1}^{\infty }\lambda _{p}B_{p} \cosh \lambda _p a \cos \lambda _{p}(y+h), \end{aligned}$$

where $B_0$ and $B_p$ are coefficients to be determined in the process of the solution.

For the determination of the function $\Phi (x,y)$, introduce the finite cosine Fourier transform of this function defined as:

20 $$\begin{aligned} \widetilde{\Phi }_{m}(y)=\int _{0}^{a}\,\Phi (x,y)\,\cos \frac{m \pi x }{a} \,dx,\quad m=0,1,2, \ldots \end{aligned}$$

The inversion formula reads:

21 $$\begin{aligned} \Phi (x,y)=\sum _{m=0}^{\infty }\frac{q_m^0}{a}\widetilde{ \,\Phi } _{m}(y)\cos \frac{m \pi x}{a}. \end{aligned}$$

Transforming Laplace’s equation and using the continuity requirements of the horizontal derivative at two vertical lines $x=0$, $x=a$ one obtains:

22 $$\begin{aligned} \frac{\partial ^2}{\partial y^2} \widetilde{\Phi }_{m}(y) - \left( \frac{m \pi }{a} \right) ^2 \widetilde{\Phi }_{m}(y) = \zeta (y) + (-1)^m \xi (y), \end{aligned}$$

with

23 $$\begin{aligned} \zeta (y)= & \, i \lambda _0 B_0 \cosh \lambda _0(y+h) + \sum _{p=1}^{\infty }\lambda _p B_p \cosh \lambda _p a \cos \lambda _p(y+h), \end{aligned}$$

24 $$\begin{aligned} \xi (y)= & \, i \lambda _0 \left( I_0 e^{-i\lambda _{0}a} - R_0 e^{i\lambda _{0}a} \right) \cosh \lambda _0(y+h) + \sum _{p=1}^{\infty }\lambda _p R_p \cos \lambda _p(y+h) \nonumber \\+ & {} C\left( \frac{a}{a^2+y^2} + \frac{a}{a^2+(y+2h)^2} \right) . \end{aligned}$$

The function multiplying *C* in the last equation may now be expanded in the complete set of functions $\{ \cosh \lambda _0 (y+h), \cos \lambda _p (y+h), p=1,2, \cdots \}$ on the interval $[-h,0]$ as:

25 $$\begin{aligned} \frac{a}{a^2+y^2} + \frac{a}{a^2+(y+2h)^2} = \beta _{0}\lambda _0 \cosh \lambda _0 (y+h) + \sum _{p=1}^{\infty } \beta _{p}\lambda _p \cos \lambda _p (y+h), \end{aligned}$$

where $\beta _{0}, \beta _{p}$ are shown in Suppl. Appendix. It may be easily verified that this function is positive, bounded and monotonic decreasing on its interval of definition. Moreover, it has zero derivative at $y=-1$, showing that the expansion can be differentiated term by term.

The transformed condition on the water surface reads:

26 $$\begin{aligned} h \frac{\partial \widetilde{\Phi }_{m}(y)}{\partial y} - \kappa \widetilde{\Phi }_{m}(y)= & \, \kappa \left[ E \frac{a}{2} \, \delta _m^{2N} + a C W_m \right] + C h \left( \frac{\pi }{8} - Q_m \right) + i \nu a P_m \nonumber \\= & \, i \nu a P_m', \, \, \text {say,} \end{aligned}$$

with

27 $$\begin{aligned} \nu = \omega h^2, \nonumber \\ W_m= & {} - \frac{1}{4} \frac{1}{m \pi } \int _{0}^{a}\, \sin \frac{m \pi x}{a} \left[ \frac{1}{x} + \frac{x}{x^2 + 4 h^2} + \frac{1}{2a - x} + \frac{2a - x}{(2a - x)^2 + 4 h^2} \right] \, dx, \end{aligned}$$

28 $$\begin{aligned} Q_m= & {} 2 a h \int _0^a \frac{a - x}{\left( x^2 + 4 h^2 \right) \left[ (2a - x)^2 + 4h^2 \right] } \cos \frac{m \pi x}{a} \, dx \end{aligned}$$

and

$$\begin{aligned} P_m' = P_m - i E \frac{\kappa }{2 \nu } \, \delta _m^{2N} - i C \frac{\kappa }{\nu } \left[ W_m + \frac{h}{\kappa a} \left( \frac{\pi }{8} - Q_m \right) \right] . \end{aligned}$$

The quantities $W_m$ and $Q_m$ tend to zero like 1/*m* as *m* increases indefinitely. The first integral in the expression for $W_m$ has a removable singularity and equals:

29 $$\begin{aligned} \int _{0}^{a}\, \frac{1}{x} \sin \frac{m \pi x}{a} \, dx = \int _{0}^{m \pi }\, \frac{1}{x} \sin x \, dx = \text {Si}(m \pi ), \end{aligned}$$

$\text {Si}(z)$ denoting the usual integral sine function in the variable *z*.

Solving Eq. (22), satisfying the condition on the water surface and applying the inverse transformation, one obtains:

30 $$\begin{aligned} \Phi (x,y)= & {} \sum _{\begin{array}{c} m=0 \end{array}}^{M} q_m^0 \frac{A_m}{\cosh \frac{m \pi h}{a}} \left( \cosh \frac{m \pi y}{a} + \frac{a}{h} \frac{1}{m \pi } \, \kappa \, \sinh \frac{m \pi y}{a} \right) \cos \frac{m \pi x}{a} \nonumber \\{} & {} + i \nu \sum _{\begin{array}{c} m=0 \end{array}}^{M} \frac{q_m^0}{m \pi } \, P_m' \, \sinh \frac{m \pi y}{a} \cos \frac{m \pi x}{a} + iB_0 \sin \lambda _0 x \cosh \lambda _0(y+h) \nonumber \\{} & {} + \frac{i}{\sin \lambda _0a} \left( B_0 \cos \lambda _0 a + I_0 e^{-i\lambda _{0}a} - R_0 e^{i\lambda _{0}a} - i C \beta _0 \right) \cos \lambda _0 x \cosh \lambda _0 (y+h) \nonumber \\{} & {} - \sum _{p=1}^{P} B_p \cosh \lambda _p a \frac{\cosh \lambda _p (a-x)}{\sinh \lambda _p a} \cos \lambda _p (y+h) \nonumber \\{} & {} - \sum _{p=1}^{P} \left( R_p + C \beta _p \right) \frac{\cosh \lambda _p x}{\sinh \lambda _p a} \cos \lambda _p (y+h), \end{aligned}$$

Under the assumption

$$\begin{aligned} \lambda _0 a = 2 N \pi , \end{aligned}$$

where *N* is the same positive integer introduced in Eq. (16), chosen large enough so as to include the non-horizontal part of the bottom and the finite support of the surface pressure excess in the region $V^0$, the term with vanishing denominator in the expression (30) will have a removable singularity if the numerator vanishes:

31 $$\begin{aligned} R_0 = B_0 + I_0 - i \beta _0 C \end{aligned}$$

and the following limit exists:

$$\begin{aligned} D= & {} \lim _{\lambda _0 a \rightarrow 2 N \pi } \, \frac{i}{\sin \lambda _0a} \left( B_0 \cos \lambda _0 a + I_0 e^{-i\lambda _{0}a} - R_0 e^{i\lambda _{0}a} - i C \beta _0 \right) \\= & {} \lim _{\lambda _0 a \rightarrow 2 N \pi } \, \frac{i}{\sin \lambda _0a} \left[ B_0 \left( \cos \lambda _0 a - 1 \right) + I_0 \left( e^{-i\lambda _{0}a} - 1 \right) + R_0 \left( e^{i\lambda _{0}a} - 1 \right) \right] . \end{aligned}$$

The limit may be taken at once to yield:

32 $$\begin{aligned} D = I_0 + R_0. \end{aligned}$$

The expresssion for $\Phi$ now simplifies to:

33 $$\begin{aligned} \Phi (x,y)= & {} \sum _{\begin{array}{c} m=0 \end{array}}^{M} q_m^0 \frac{A_m}{\cosh \frac{m \pi h}{a}} \left( \cosh \frac{m \pi y}{a} + \frac{a}{h} \frac{1}{m \pi } \, \kappa \, \sinh \frac{m \pi y}{a} \right) \cos \frac{m \pi x}{a} \nonumber \\{} & {} + i \nu \sum _{\begin{array}{c} m=0 \end{array}}^{M} \frac{q_m^0}{m \pi } \, P_m' \, \sinh \frac{m \pi y}{a} \cos \frac{m \pi x}{a} + \left( I_0 + R_0 \right) \cosh \lambda _0 (y+h) \cos \lambda _0 x \nonumber \\{} & {} + iB_0 \sin \lambda _0 x \cosh \lambda _0(y+h) -\sum _{p=1}^{P} B_p \cosh \lambda _p a \frac{\cosh \lambda _p (a-x)}{\sinh \lambda _p a} \cos \lambda _p (y+h) \nonumber \\{} & {} - \sum _{p=1}^{P} \left( R_p + C \beta _p \right) \frac{\cosh \lambda _p x}{\sinh \lambda _p a} \cos \lambda _p (y+h), \end{aligned}$$

The function $\displaystyle \cosh \frac{2N \pi y}{a}$ is now expanded in the complete set of functions $\{ \cosh \lambda _0 (y+h), \cos \lambda _p (y+h), p=1,2, \cdots \}$ on the interval $[-h,0]$ as:

34 $$\begin{aligned} \cosh \frac{2N \pi y}{a}= & {} I_{2N,0} \cosh \lambda _0 (y+h) + \sum _{p=1}^{\infty } I_{2N,p} \cos \lambda _p (y+h), \end{aligned}$$

where $I_{m0}, I_{mp}$ are shown in Suppl. Appendix.

### Continuity of the solution at $x=a$

The continuity requirement of the velocity potential at $x=a$ yields

35 $$\begin{aligned}{} & {} \sum _{\begin{array}{c} m=0 \end{array}}^{M} (-1)^m q_m^0 \frac{A_m}{\cosh \frac{m \pi h}{a}} \left( \cosh \frac{m \pi y}{a} + \frac{a}{h} \frac{1}{m \pi } \, \kappa \, \sinh \frac{m \pi y}{a} \right) \nonumber \\{} & {} \qquad + i \nu \sum _{\begin{array}{c} m=0 \end{array}}^{M} (-1)^m \, \frac{q_m^0}{m \pi } \, P_m' \, \sinh \frac{m \pi y}{a} + E I_{2N,0} \cosh \lambda _0 (y+h) \nonumber \\{} & {} \qquad + \sum _{p=1}^{P} \left[ E I_{2N,p} - R_{p} - \left( B_p + R_p e^{-\lambda _{p}a} + C \beta _p \right) \coth \lambda _p a \right] \cos \lambda _p (y+h) = 0. \end{aligned}$$

By orthogonality, using Eqs. (31, 32) and after some manipulations, one obtains:

36 $$\begin{aligned} C= & \, c_1 + c_2 E + \frac{1}{\rho _0 \kappa } \sum _{\begin{array}{c} m=0 \end{array}}^{\infty } (-1)^m q_m^0 G_m \frac{A_m}{\cosh \frac{m \pi h}{a}} \end{aligned}$$

37 $$\begin{aligned} R_0= & \, r_1 + r_2 E + B_0 - \frac{i \beta _0}{\rho _0 \kappa } \sum _{\begin{array}{c} m=0 \end{array}}^{\infty } (-1)^m q_m^0 G_m \frac{A_m}{\cosh \frac{m \pi h}{a}} \end{aligned}$$

38 $$\begin{aligned} R_p= & \, s^{(1)}_p + s^{(2)}_p E - \frac{\coth \lambda _p a}{1 + e^{- \lambda _p a}\coth \lambda _p a} B_p \nonumber \\{} & {} + \frac{1}{1 + e^{- \lambda _p a} \coth \lambda _p a} \sum _{\begin{array}{c} m=0 \end{array}}^{\infty } (-1)^m q_m^0 \left[ G_{mp} + \frac{\rho _p \kappa - \beta _p \coth \lambda _p a}{\rho _0 \kappa } G_m \right] \frac{A_m}{\cosh \frac{m \pi h}{a}} \nonumber \\ \end{aligned}$$

in terms of the incident wave amplitude $I_0$ and the unknown coefficients

$$\begin{aligned} \{ E, B_0, B_p, A_m \}. \end{aligned}$$

Ultimately, the unknown coefficients will be determined approximately from the satisfaction of the bottom boundary condition. The introduced notation is listed in Suppl. Appendix.

Singular solutions with prescribed strength *C*, or regular solutions with $C = 0$ may be obtained from the above formulae, in which case one gets an additional constraint between the unknown coefficients of the problem. The regular solution, however, may conceal important physical features of the considered problem ( Cf. Wehausen and Laitone^7^, p. 537).

The total velocity potential in the region *V* is:

39 $$\begin{aligned} \Phi ^0(x,y)= & \, \sum _{\begin{array}{c} m=0 \end{array}}^{M} q_m^0 \frac{A_m}{\cosh \frac{m \pi h}{a}} \left( \cosh \frac{m \pi y}{a} + \frac{a}{h} \frac{1}{m \pi } \, \kappa \, \sinh \frac{m \pi y}{a} \right) \cos \frac{m \pi x}{a} \nonumber \\ & +  {} i \nu \sum _{\begin{array}{c} m=0 \end{array}}^{M} \frac{q_m^0}{m \pi } \, P_m' \, \sinh \frac{m \pi y}{a} \cos \frac{m \pi x}{a} + \left( I_0 + R_0 \right) \cosh \lambda _0 (y+h) \cos \lambda _0 x \nonumber \\{} & {} + iB_0 \sin \lambda _0 x \cosh \lambda _0(y+h) - \sum _{p=1}^{P} B_p \cosh \lambda _p a \frac{\cosh \lambda _p (a-x)}{\sinh \lambda _p a} \cos \lambda _p (y+h) \nonumber \\{} & {} - \sum _{p=1}^{P} \left( R_p e^{-\lambda _{p}a} + C \beta _p \right) \frac{\cosh \lambda _p x}{\sinh \lambda _p a} \cos \lambda _p (y+h) \nonumber \\{} & {} + E \, \cosh \frac{2N \pi y}{a} \cos \frac{2N \pi x}{a} + C \, Z(x,y). \end{aligned}$$

## Determination of the unknown coefficients

Prior to the application of the impermeability condition on the bottom, the expressions for the velocity potential $\Phi ^0$ and the stream function $\Psi ^0$ are re-written in a form that exhibits their dependence on the unknown coefficients:

40 $$\begin{aligned} \Phi ^0(x,y) = \Phi ^{(0)}_0(x,y) + \sum _{\begin{array}{c} m=0 \end{array}}^{\infty } q_m^0 A_m \Phi ^{(1)}_m(x,y) + \sum _{\begin{array}{c} p=0 \end{array}}^{\infty } B_p \Phi ^{(2)}_p(x,y) + E \Phi ^{(3)}(x,y) \end{aligned}$$

and

41 $$\begin{aligned} \Psi ^0(x,y) = \Psi ^{(0)}_0(x,y) + \sum _{\begin{array}{c} m=0 \end{array}}^{\infty } q_m^0 A_m \Psi ^{(1)}_m(x,y) + \sum _{\begin{array}{c} p=0 \end{array}}^{\infty } B_p \Psi ^{(2)}_p(x,y) + E \Psi ^{(3)}(x,y). \end{aligned}$$

The different functions are listed in Suppl. Appendix.

It remains now to determine the constants $C, E, B_p, \,( p=0,1,2, \ldots )$ and $A_m, \, (m=0,1,2, \ldots )$ by satisfying the boundary impermeability condition $\Psi ^0 = 0$ on the bottom by a suitable boundary method. We note two methods:

- Boundary collocation: Satisfying the condition at points $P_r, r=1,2,...,R$ adequately chosen on the bottom line *L* produces *R* linear algebraic equations in a finite number of the unknowns $B_p, \, p=0,1,2, \ldots ,P$ and $A_m, \, m=0,1,2, \ldots ,M$. The absolute error in satisfying the boundary condition on the bottom line is defined as
  42 $$\begin{aligned} E_1 = \max _Q \left| \psi ^0(Q) \right| , \qquad Q \in L. \end{aligned}$$
- A boundary Fourier expansion method: Let $\theta , \, 0 \le \theta \le \Theta$ be a running parameter for the bottom curve *L* in the region $V^0$. Then $\Psi ^0 = \Psi ^0(\theta ) = 0$ on *L*. Expand this function in a Fourier series on $[0,\Theta ]$. Then Fourier coefficients of this function must vanish. This provides a set of linear algebraic equations for the unknown coefficients. The error here is defined as:
  43 $$\begin{aligned} E_2 = \frac{1}{\vert L \vert } \int _L \vert \Psi ^0(\theta ) \vert ^2 \, d \theta . \end{aligned}$$

Due to the existence of singularities in the function of the velocity potential and its derivatives at the origin, it is believed that the application of the boundary integral method (Cf. the boundary Fourier expansion method^43^), is appropriate for better results. It is thus clear that the method deals in a unified way with any shape of the bottom curve. This is an important feature and an advantage of the present method.

## Computational aspects

When carrying out computations, the infinite series over *m* and *p* in the solutions must be truncated. The same holds for the Fourier representation of the surface pressure excess function $P_0(x)$ which will be assumed to have only $K+1$ terms. Proper limits must then be taken. The divergent series for $\rho _0$ and $\rho _p$ (see Suppl. Appendix) must be re-written as:

$$\begin{aligned} \rho _0= & \, \frac{G_M}{J_0} \sum _{\begin{array}{c} m=0 \end{array}}^{M} (-1)^m \, \frac{q_m^0}{m \pi } \, \left[ W_m + \frac{h}{\kappa a} \left( \frac{\pi }{8} - Q_m \right) \right] \, \frac{J_{m0}}{G_M}, \\ \rho _p= & \, \frac{G_M}{J_p} \sum _{\begin{array}{c} m=0 \end{array}}^{M} (-1)^m \, \frac{q_m^0}{m \pi } \, \left[ W_m + \frac{h}{\kappa a} \left( \frac{\pi }{8} - Q_m \right) \right] \, \frac{J_{mp}}{G_M} \end{aligned}$$

The Eqs. (36), (31) and (38) now assume the form:

44 $$\begin{aligned} C= & \, c_1 + c_2 E + \frac{1}{\kappa } \, \frac{\displaystyle \sum _{\begin{array}{c} m=0 \end{array}}^{M} (-1)^m q_m^0 \frac{G_m}{G_M} \frac{A_m}{\cosh \frac{m \pi h}{a}}}{\frac{\rho _0}{G_M}} \end{aligned}$$

45 $$\begin{aligned} R_0= & \, r_1 + r_2 E + B_0 - \frac{i \beta _0}{\kappa } \, \frac{\displaystyle \sum _{\begin{array}{c} m=0 \end{array}}^{M} (-1)^m q_m^0 \frac{G_m}{G_M} \frac{A_m}{\cosh \frac{m \pi h}{a}}}{\frac{\rho _0}{G_M}} \end{aligned}$$

46 $$\begin{aligned} R_p= & \, s^{(1)}_p + s^{(2)}_p E - \frac{\coth \lambda _p a}{1 + e^{- \lambda _p a}\coth \lambda _p a} B_p \nonumber \\{} & {} + \frac{1}{1 + e^{- \lambda _p a}\coth \lambda _p a} \, \sum _{\begin{array}{c} m=0 \end{array}}^{M} (-1)^m q_m^0 \left[ \frac{G_{mp}}{\cosh \frac{m \pi h}{a}} + \frac{1}{\kappa } \, \frac{\frac{\kappa \rho _p}{G_M} - \frac{\beta _p}{G_M} \coth \lambda _p a}{\frac{\rho _0}{G_M}} \frac{G_m}{\cosh \frac{m \pi h}{a}} \right] A_m \end{aligned}$$

The coefficients appearing in these last equations are expressed as:

$$\begin{aligned} c_1= & {} \frac{i \nu }{\kappa } \, \frac{ \displaystyle \sum _{\begin{array}{c} m=0 \end{array}}^{M} (-1)^m \, \frac{q_m^0}{m \pi } \frac{J_{m0}}{J_0} \, \frac{P_m}{G_M}}{\frac{\rho _0}{G_M}}, \\ c_2= & {} \frac{1}{\kappa \rho _0} \left( I_{2N,0} + \frac{\kappa }{2 N \pi } \frac{J_{2N,0}}{J_0} \right) \simeq 0. \\ s^{(1)}_p= & {} \frac{i \nu }{1 + e^{-\lambda _{p}a} \coth \lambda _p a} \left[ \frac{\kappa \frac{\rho _p}{G_M} - \frac{\beta _p}{G_M} \coth \lambda _p a }{\kappa \frac{\rho _0}{G_M}} \sum _{\begin{array}{c} m=0 \end{array}}^{K} (-1)^m \, \frac{q_m^0}{m \pi } \frac{J_{m0}}{J_0} \, P_m \right. \\{} & {} + \left. \sum _{\begin{array}{c} m=0 \end{array}}^{K} (-1)^m \, \frac{q_m^0}{m \pi } \, P_m \, \frac{J_{mp}}{J_p} \right] , \\ s^{(2)}_p= & {} \frac{1}{1 + e^{-\lambda _{p}a} \coth \lambda _p a} \left[ \frac{\kappa \frac{\rho _p}{G_M} - \frac{\beta _p}{G_M} \coth \lambda _p a }{\kappa \frac{\rho _0}{G_M}} \left( I_{2N,0} + \frac{\kappa }{2 N \pi } \frac{J_{2N,0}}{J_0} \right) \right. \\{} & {} + \left. \left( I_{2N,p} + \frac{\kappa }{2N \pi } \frac{J_{2N,p}}{J_p} \right) \right] \end{aligned}$$

## Trapping of waves

Formula (31) obtained for the reflection coefficient allows to treat cases where the incident wave is totally absorbed due surface pressure excess. For this, it suffices to set $R_0=0$ in that formula to yield:

47 $$\begin{aligned} r_1 + r_2 E + B_0 + \frac{i \beta _0}{\rho _0} \frac{h}{a} \sum _{\begin{array}{c} m=0 \end{array}}^{\infty } (-1)^m q_m^0 G_m A_m = 0. \end{aligned}$$

This equation will now be considered to involve the unknown constants $P_m$, to be determined together with the other unknown coefficients of the problem.

For the particular choice of a constant pressure excess over the interval [0, *a*], all the coefficients $P_m$ vanish, with the exception of the (unknown) coefficient $P_0$. This latter may be found, together with the other coefficients, from the impermeability condition on the bottom curve together with the reduced equation.

Other cases of trapping may be found if all the $P_m$’s, except a finite number of them, vanish identically.

## Progressive wave against a cliff with horizontal bottom

**Figure 2:**
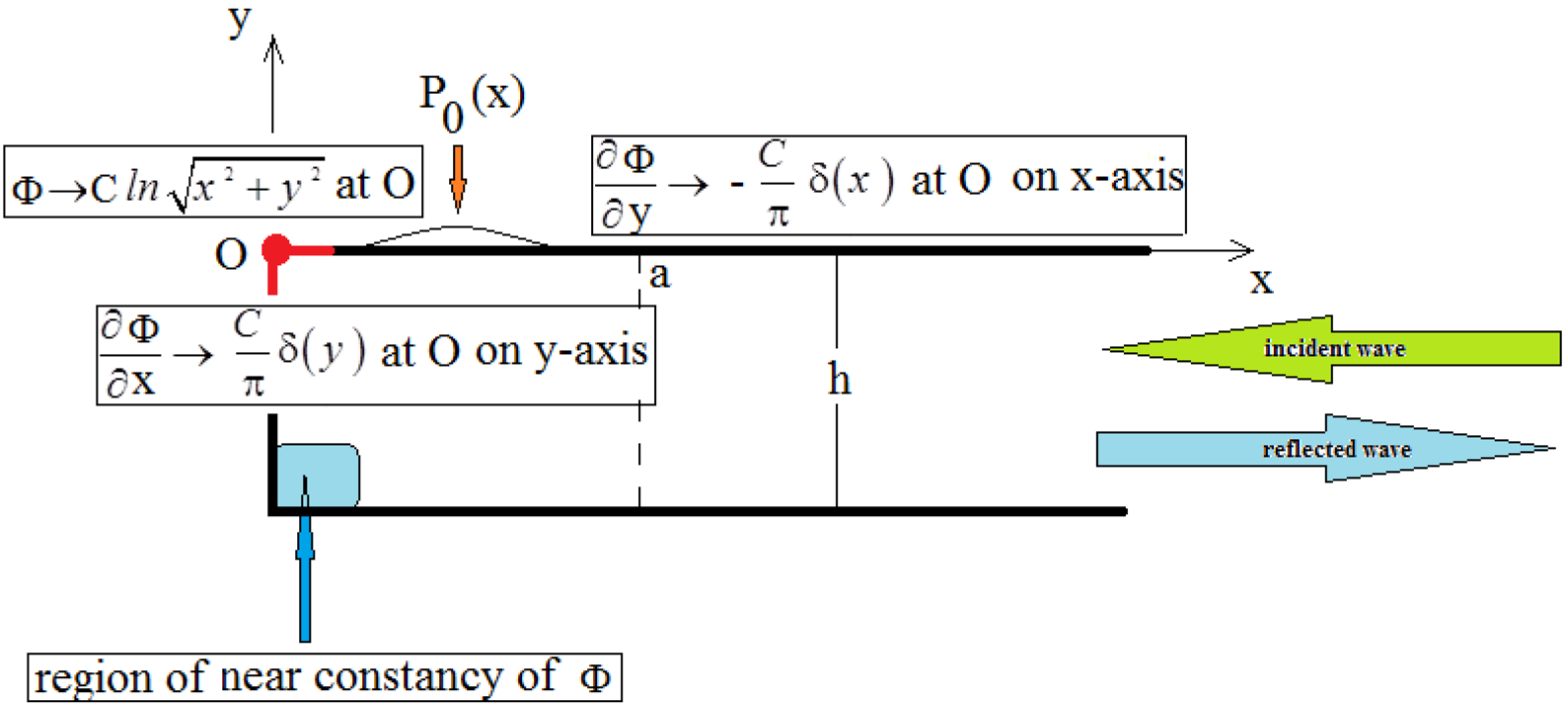
Solution regions for a cliff with horizontal bottom.

For a progressive wave against a cliff with horizontal bottom as in Fig. 2, the coefficients $B_0, B_1, B_2, \cdots$ may be calculated from the condition of vanishing of the horizontal derivative of the velocity potential $\Phi ^0$ at the cliff as follows:

48 $$\begin{aligned} i\lambda _{0} B_0 \cosh \lambda _{0}(y+h) + \sum _{p=1}^{\infty }\lambda _{p}B_{p} \cosh \lambda _p a \cos \lambda _{p}(y+h) = -\frac{C}{\pi } \delta (y), \end{aligned}$$

hence by integration using orthogonality:

$$\begin{aligned} B_0 = \frac{i C}{2 \pi } \frac{\cosh \lambda _{0} h}{\lambda _0 J_0 h}, \quad B_p = \frac{i C}{2 \pi } \frac{\cos \lambda _{p} h}{\lambda _0 J_0 h}. \end{aligned}$$

On the other hand, as shown above:

$$\begin{aligned} D=I_0 + R_0. \end{aligned}$$

Then the coefficients $A_m, m=0,1,2, \ldots$ are determined from the satisfaction of the bottom condition by equating to zero the vertical derivative of the velocity potential $\Phi ^0$ as follows:

$$\begin{aligned} 0= & {} \frac{h}{a} \sum _{\begin{array}{c} m=0 \end{array}}^{\infty } q_m^0 \frac{m \pi }{a} A_m \left( - \sinh \frac{m \pi h}{a} + \frac{a}{h} \frac{1}{m \pi } \, \kappa \, \cosh \frac{m \pi h}{a} \right) \cos \frac{m \pi x}{a} \\{} & {} + \nu \sum _{\begin{array}{c} m=0 \end{array}}^{\infty } \frac{q_m^0}{m \pi } \, P_m' \, \frac{m \pi }{a} \cosh \frac{m \pi h}{a} \cos \frac{m \pi x}{a} \\{} & {} - E \, \frac{2N \pi }{a} \sinh \frac{2N \pi h}{a} \cos \frac{2N \pi x}{a} - C \, \frac{h}{x^2 + h^2}. \end{aligned}$$

Make a Fourier cosine expansion

49 $$\begin{aligned} \frac{h}{x^2 + h^2}=\sum _{m=0}^{\infty }\frac{q_m^0}{a}M_{m} \cos \frac{m \pi x}{a}, \end{aligned}$$

so that:

50 $$\begin{aligned} M_{m} =\int _{0}^{a}\,\frac{h}{x^2 + h^2}\,\cos \frac{m \pi x }{a} \,dx,\,m=0,1,2, \ldots . \end{aligned}$$

The condition on the bottom becomes:

$$\begin{aligned} 0= & {} \frac{h}{a} \sum _{\begin{array}{c} m=0 \end{array}}^{\infty } q_m^0 \frac{m \pi }{a} A_m \left( - \sinh \frac{m \pi h}{a} + \frac{a}{h} \frac{1}{m \pi } \, \kappa \, \cosh \frac{m \pi h}{a} \right) \cos \frac{m \pi x}{a} \\{} & {} + \nu \sum _{\begin{array}{c} m=0 \end{array}}^{\infty } \frac{q_m^0}{m \pi } \, P_m' \, \frac{m \pi }{a} \cosh \frac{m \pi h}{a} \cos \frac{m \pi x}{a} \\{} & {} - E \, \frac{2N \pi }{a} \sinh \frac{2N \pi h}{a} \cos \frac{2N \pi x}{a} - C \, \sum _{m=0}^{\infty }\frac{q_m^0}{a}M_{m} \cos \frac{m \pi x}{a}. \end{aligned}$$

For $m = 2N$ the above equation yields the value of the coefficient *E* from the relation:

$$\begin{aligned} \left( 1 - \frac{a \kappa }{2 N \pi } \right) E= & {} \frac{\nu }{N \pi } P_{2N} \\{} & {} + C \left[ \frac{ h}{2 N \pi ^2} \{ 1 - \frac{2 a \pi \kappa }{ 2N \pi } \, \text {Si}(2N \pi ) \} \coth \frac{2N \pi h}{a} - \frac{ M_{2N}}{N \pi \sinh \frac{2N \pi h}{a}} \right] . \end{aligned}$$

For the other values of *m*, one gets the values of the constants $A_m$ in terms of the other unknown coefficients from the relations:

$$\begin{aligned} A_m \left( \frac{h}{a} m \pi \tanh \frac{m \pi h}{a} - \kappa \, \right) = \nu P_m' - C \frac{M_{m}}{\cosh \frac{m \pi h}{a}}. \end{aligned}$$

Equations (36) and (31) now serve to determine the two constants *C* and $R_0$. Finally, the remaining constant $A_{2N}$ may be obtained by a limiting procedure from the expression of $A_m$:

$$\begin{aligned} A_{2N} = \sinh ^2 \frac{2N \pi h}{a} \, \, \frac{\nu \left( 1 + \kappa P_{2n} + 2 N Q_{2N} \right) + \frac{C h}{2 \pi \nu } \left( 1 + \kappa - 2 \kappa \pi \tanh \frac{2N \pi h}{a} \, \text {Si} (2N \pi ) \right) }{\kappa \left( \kappa + \sinh ^2 \frac{2N \pi h}{a} \right) }, \end{aligned}$$

with

$$\begin{aligned} Q_{2N} = - \frac{1}{\rho g h} \frac{\pi }{a} \int _0^a x P_0(x) \sin \frac{2N \pi x}{a} \, dx. \end{aligned}$$

Thus the problem of wave propagation against a cliff finds here an exact solution with logarithmic singularity for the velocity potential function at the intersection of the water surface mean position with the cliff.

For a constant pressure over the interval [0, *a*], one has $P_0 = 1, \, P_m = 0, m=1,2, \cdots$.

The case when the pressure function is a delta function concentrated at $x_0 (< a)$ was noted by Stoker^6^. This case is important for two reasons: (i) A general distribution of the surface pressure can be replaced by a superposition of concentrated distributions; (ii) When the surface pressure distribution is extremely complicated, or even unknown, and interest is mainly focused on the asymptotic behavior of the solution. Here:

$$\begin{aligned} P_m = \cos \frac{m \pi x_0}{a}, \quad \forall m \ge 0. \end{aligned}$$

## Numerical results

We have considered three types of beaches for the determination of the system of streamlines. Here we have set the surface excess pressure equal to zero. For the calculations, we have taken

$$\begin{aligned} \kappa = 1.5, \quad N=1, \quad a=3.874. \end{aligned}$$

This value for $\kappa$ shows that the conditions for shallow water theory are not satisfied.

The number of collocation nodes was taken so as to achieve a square matrix. Two methods of solution, the Gaussian elimination and the least squares yielded the same results. The best results for all the three considered cases were obtained for the truncation parameters:

$$\begin{aligned} M = 25, \quad P \le 46, \quad R = 32. \end{aligned}$$

These choices made the jump $\delta$ between the values of the stream function on both sides of the vertical line $x=a$ less than the value 0.00045.

**Figure 3:**
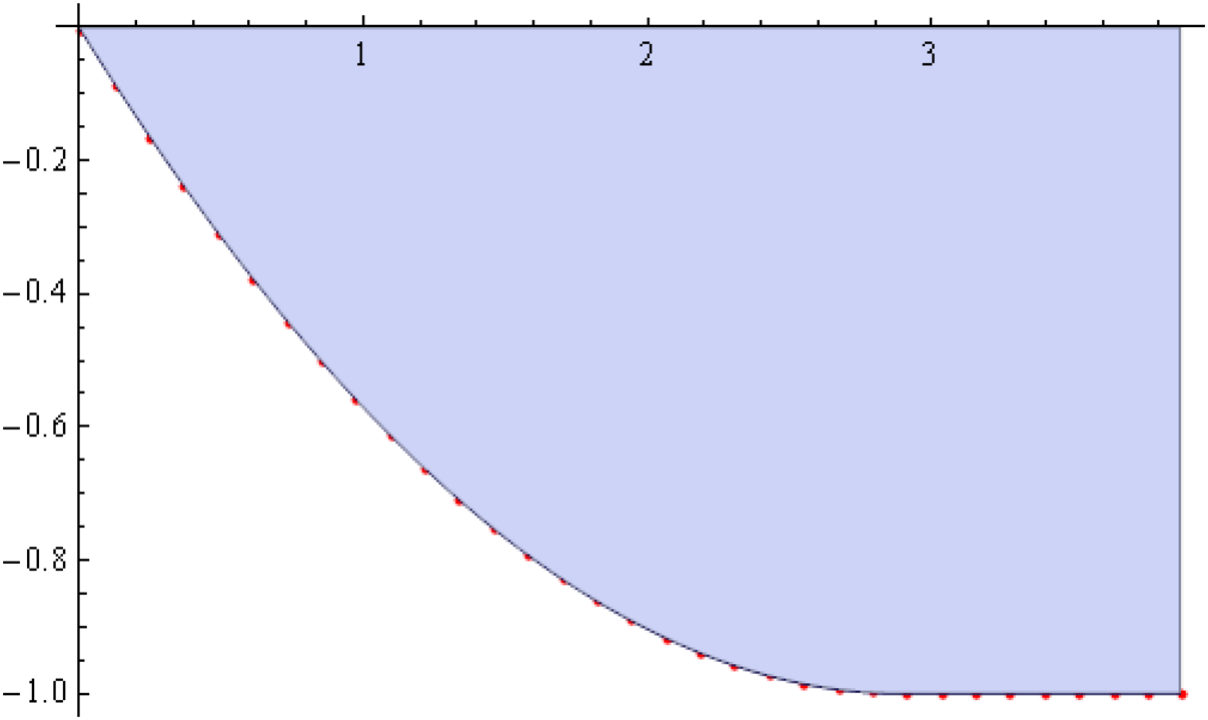
The parabolic beach and the collocation nodes.

**Figure 4:**
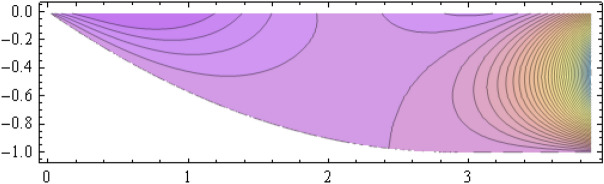
The streamlines for the parabolic beach in the region $V^0$.

**Figure 5:**
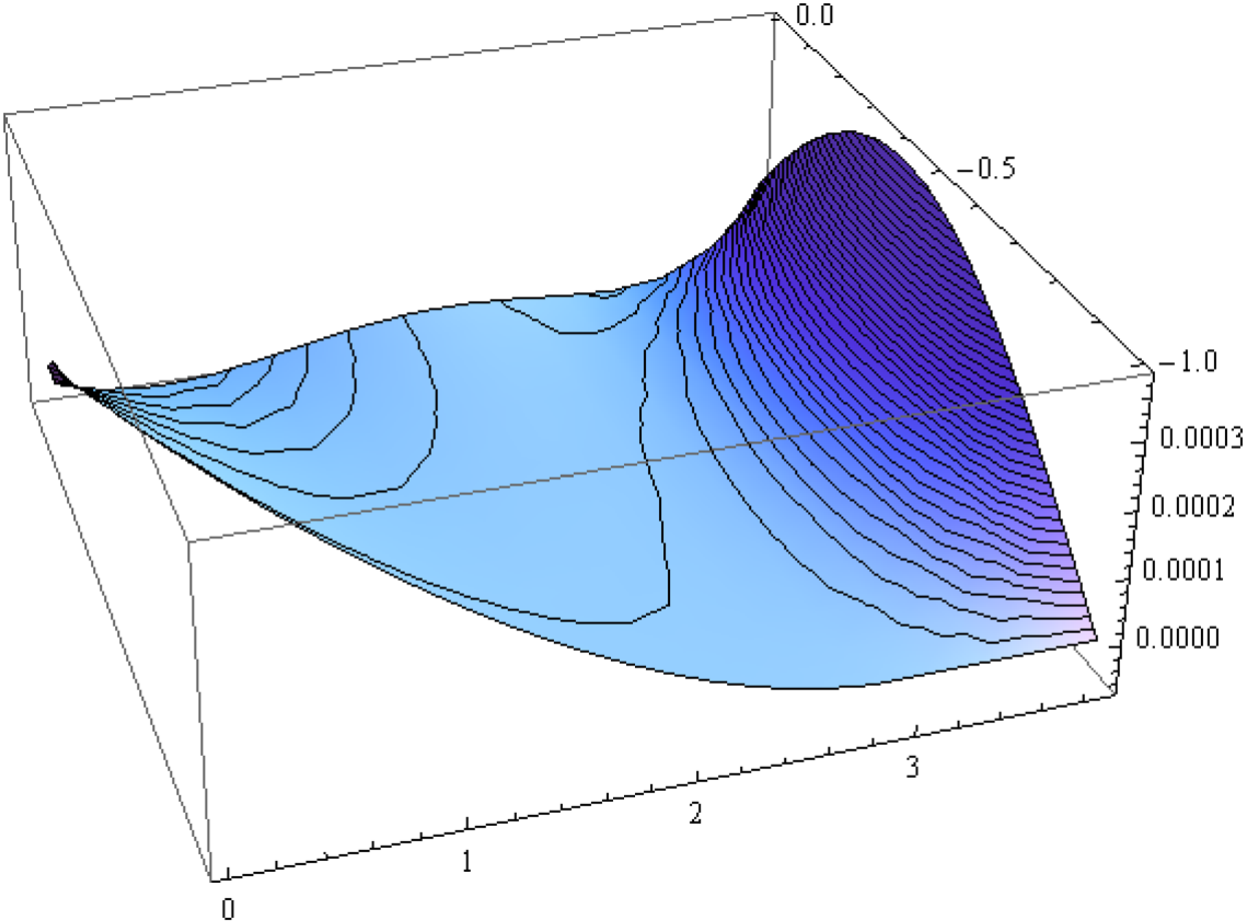
Streamfunction for the parabolic beach in the region $V^0$.

**Figure 6:**
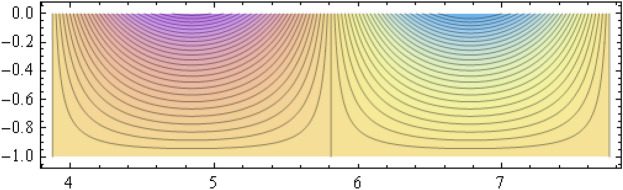
Streamlines for the parabolic beach in the region $a \le x \le 2a$.

**Figure 7:**
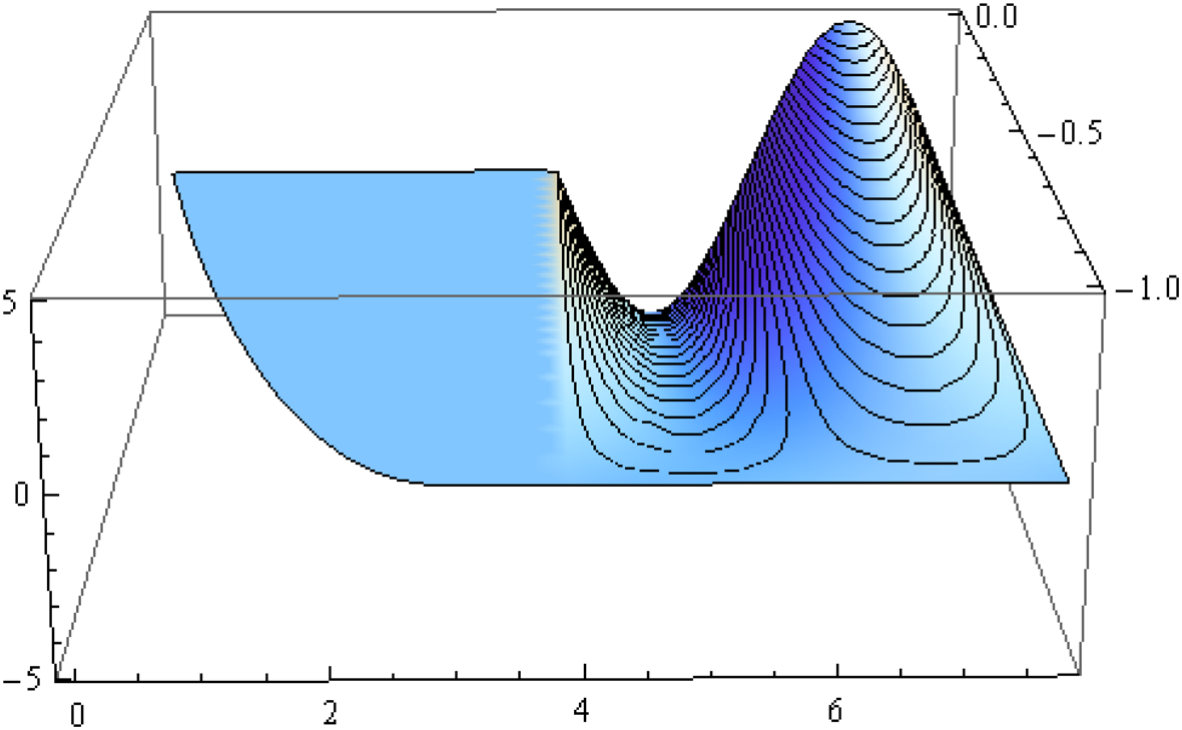
The streamfunction for the parabolic beach for $0.01 \le x \le 2a$.

**Figure 8:**
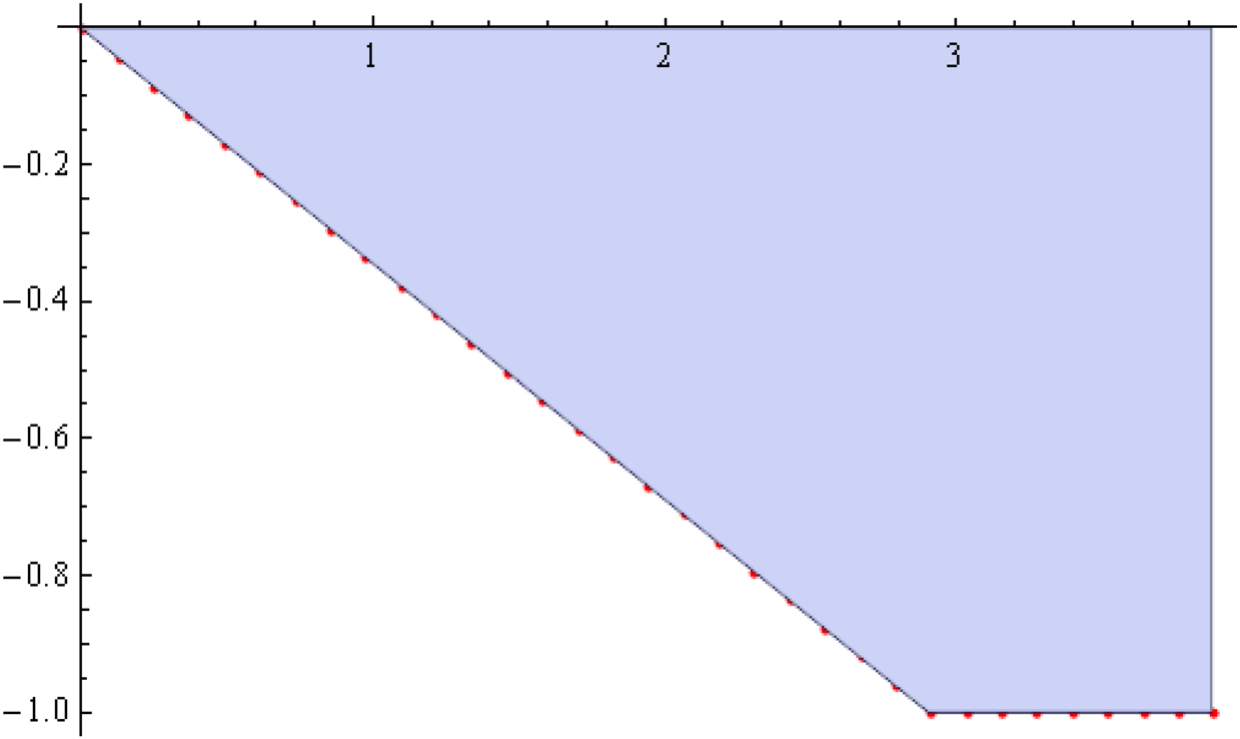
The uniformly sloping beach and the collocation nodes.

**Figure 9:**
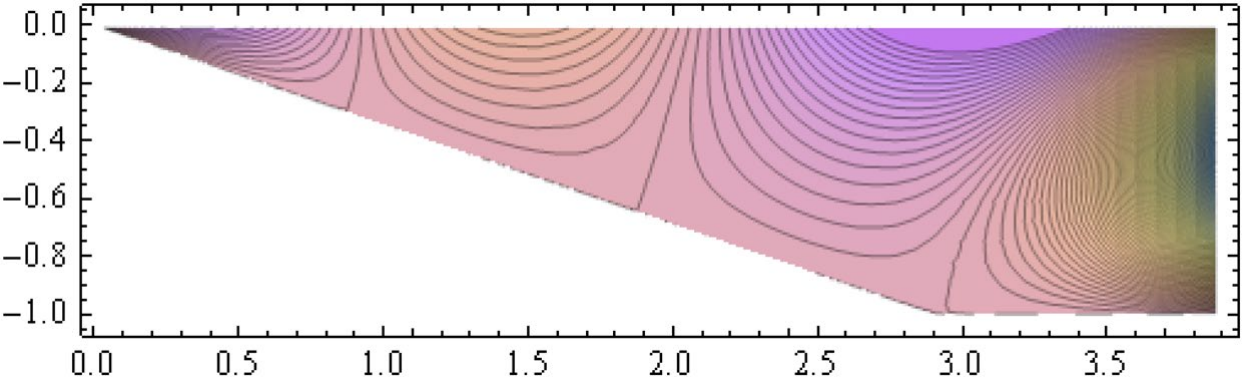
The streamlines for the uniformly sloping beach in the region $V^0$.

**Figure 10:**
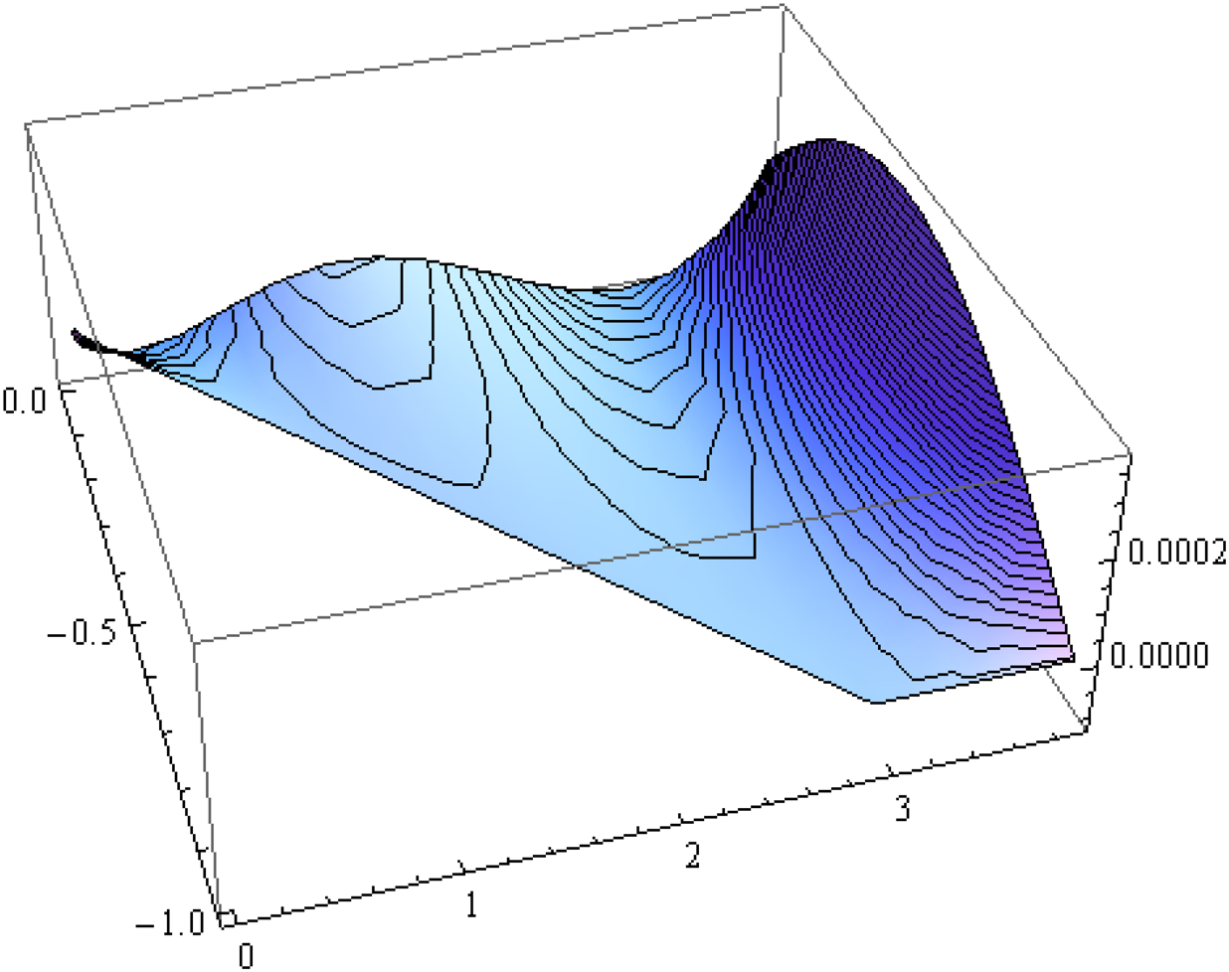
Streamfunction for the uniformly sloping beach in the region $V^0$.

**Figure 11:**
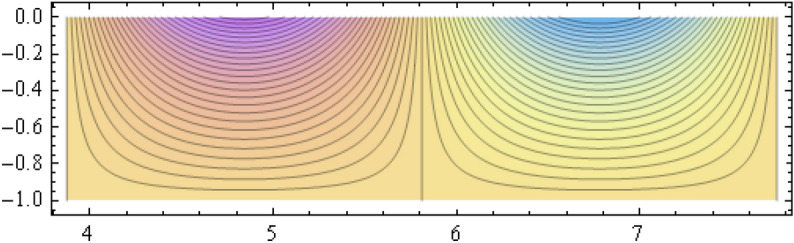
Streamlines for the uniformly sloping beach in the region for $a \le x \le 2a$.

**Figure 12:**
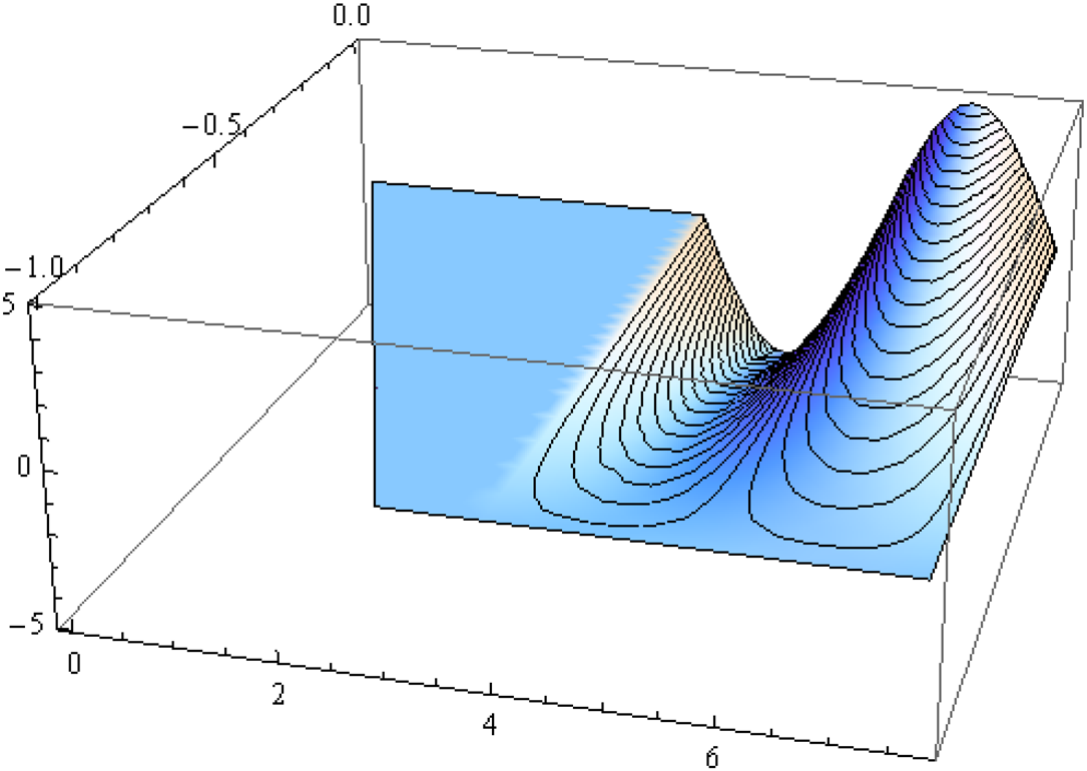
The streamfunction for the uniformly sloping beach for $0.01 \le x \le 2a$.

**Figure 13:**
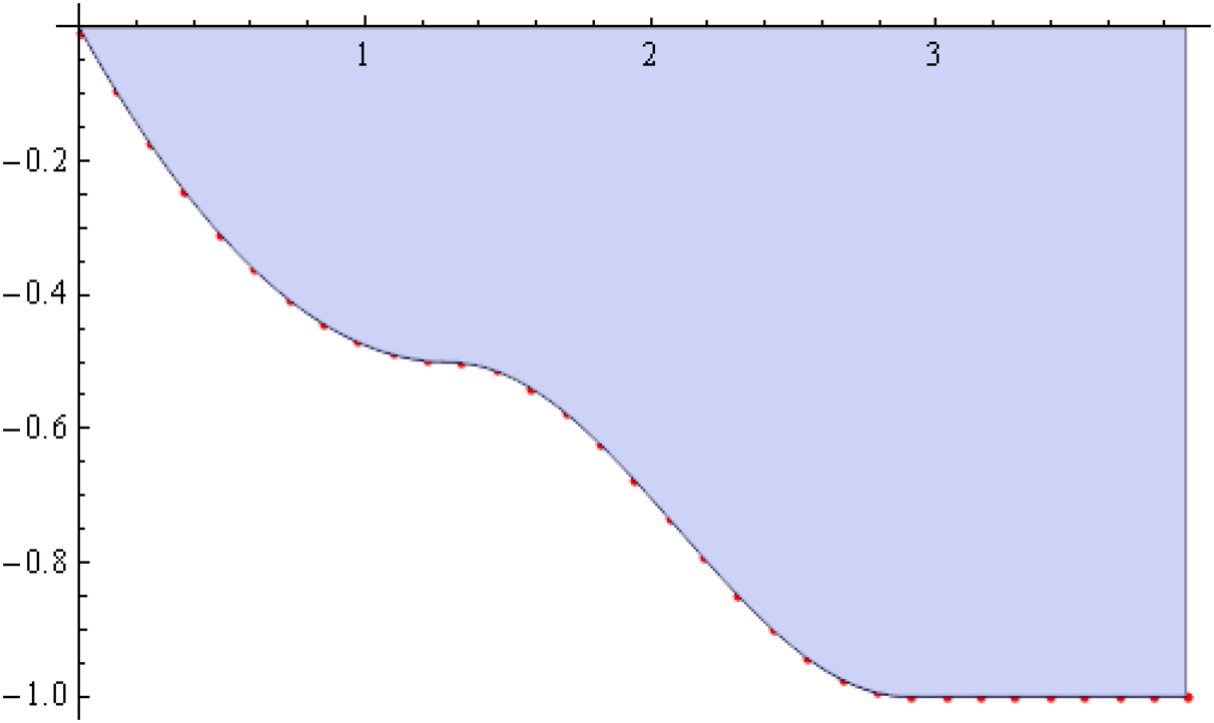
The shelf-type beach and the collocation nodes.

**Figure 14:**
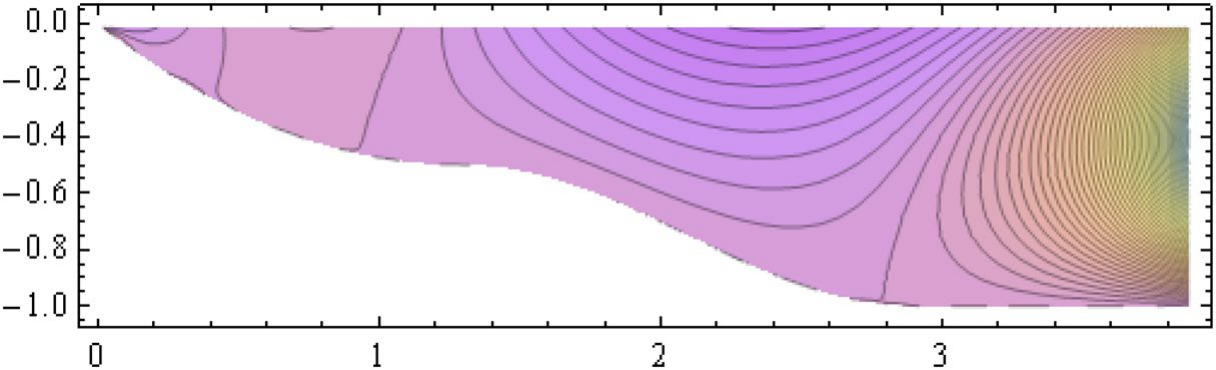
The streamlines for the shelf-type beach in the region $V^0$.

**Figure 15:**
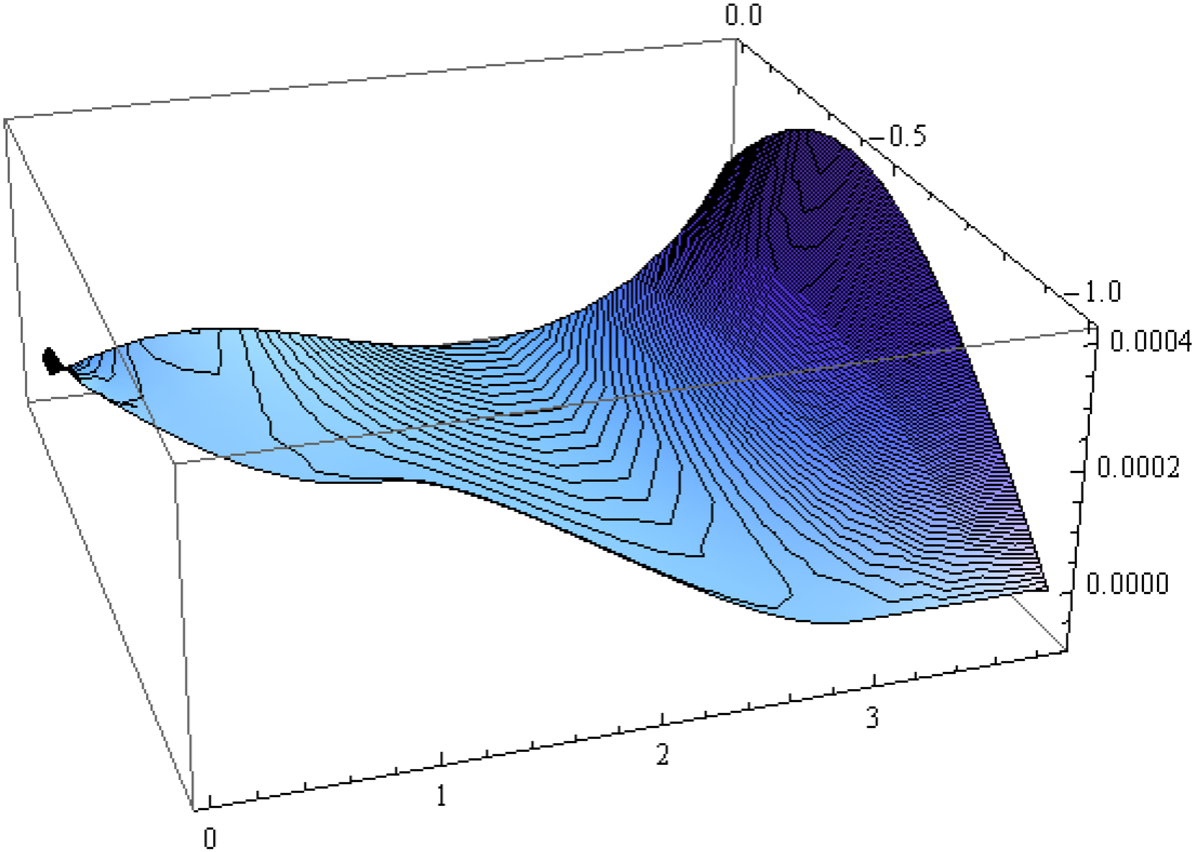
Streamfunction for the shelf-type beach in the region $V^0$.

**Figure 16:**
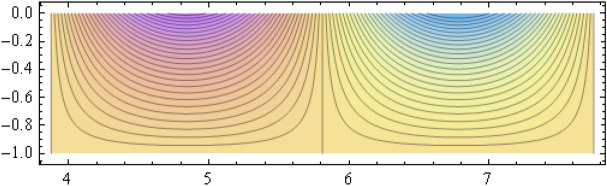
Streamlines for the shelf-type beach in the region $a \le x \le 2a$.

**Figure 17:**
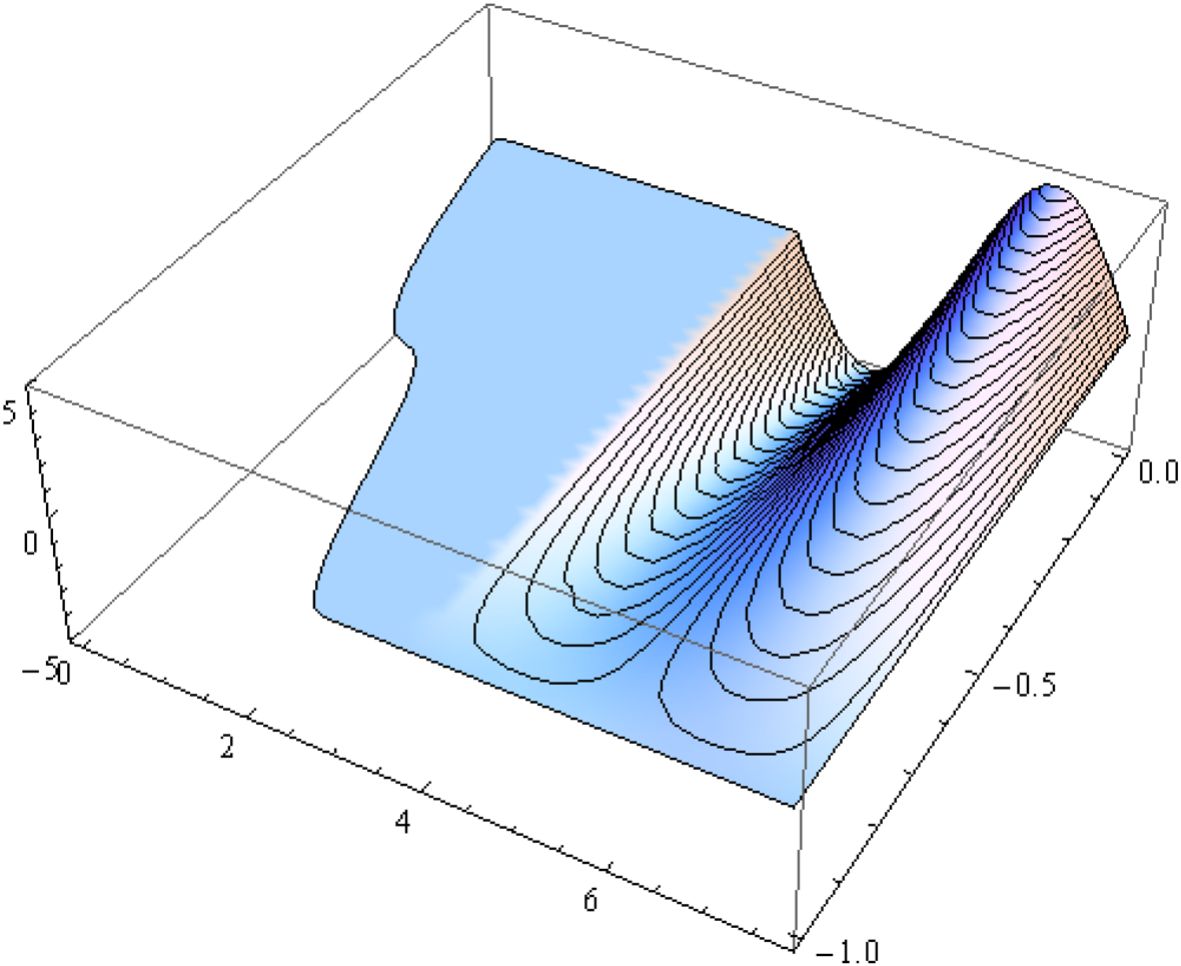
The streamfunction for the shelf-type beach for $0.01 \le x \le 2a$.

- The parabolic beach. The shape of the beach is described by the function
  51 $$\begin{aligned} k(x)= {\left\{ \begin{array}{ll} \frac{16h}{9a^2} \left( x - \frac{3a}{4} \right) ^2 - h , \quad 0 \le x \le \frac{3a}{4},\\ -h, \quad x > \frac{3a}{4}, \end{array}\right. } \end{aligned}$$
  with initial inclination $\simeq 41^{\circ }$. The shape of the beach and the collocation nodes are shown in Fig. 3. The streamlines for the parabolic beach in the region $V^0$ are illustrated in Fig. 4. The 3*D*-plot of the streamfunction in the region $V^0$ is shown in Figure 5. The streamlines for the parabolic beach in the region $a \le x \le 2a$ appear in Fig. 6, while the 3*D*-plot of the streamfunction in the region $0.01 \le x \le 2a$ is shown in Fig. 7.
- The uniformly sloping beach. The shape of the beach is described by the function
  52 $$\begin{aligned} k(x)= {\left\{ \begin{array}{ll} \frac{16h}{9a^2} \left( x - \frac{3a}{4} \right) ^2 - h , \quad 0 \le x \le \frac{3a}{4},\\ -h, \quad x > \frac{3a}{4}, \end{array}\right. } \end{aligned}$$
  with initial inclination $\simeq 19^{\circ }$. Figures 8, 9, 10, 11 and 12 illustrate the same flow characteristics as for the previous case.
- The shelf-type beach. The shape of the beach is described by the function
  53 $$\begin{aligned} k(x)= {\left\{ \begin{array}{ll} \frac{9h}{2a^2} \left( x - \frac{a}{3} \right) ^2 - \frac{h}{2} , \quad 0 \le x \le \frac{a}{3},\\ - \frac{3h}{4} + \frac{h}{4} \cos \left( \frac{12 \pi }{5a} x - \frac{12 \pi }{15} \right) , \quad \frac{a}{3} < x \le \frac{3a}{4}, \\ -h, \quad x > \frac{3a}{4}, \end{array}\right. } \end{aligned}$$
  with initial inclination $\simeq 37^{\circ }$. We have shown in Figs. 13, 14, 15, 16 and 17 the different flow characteristics for the shelf-shaped beach.

All over the semi-infinite channel, the system of stremlines forms cells. This is due to the harmonic time dependence of the incident wave. In that part of the channel with $x \ge a$ where the bottom is horizontal, there establishes a system of streamlines composed of the bottom line $\psi = 0$ and a denumerable number of vertical lines at $x_r = (r+1) \frac{a}{2}, r =1, 2, \ldots$ connecting the free surface to the bottom line. These streamlines form cells inside which all the other streamlines are distributed, all of them ultimately reaching to the free surface. The pattern consists of repeated units, each formed of two consecutive cells. These cells are shown for all three types of beaches on Figs. 6, 11 and 16 for that portion of the channel for which $a \le x \le 2a$. As time goes on, an animation program has shown that this system of cells moves as a whole to the right with uniform velocity $\frac{\omega }{\lambda _0}$.

Animation experiments have revealed that a system of standing waves establishes in the initial, left part of the channel for which $x < a$, with small vertical oscillations of the system of streamlines. In the right part of the channel, there are progressive waves propagating toward infinity. In between, there exists a buffer zone encompassing the vertical line and consisting of one cell performing horizontal oscillations and splitting periodically. Taking into account the surface excess pressure will lead to the modification of the streamlines, and the values taken by the streamfunction on these streamlines.

## Conclusions

Using a method relying on finite Fourier transform, an approximate spectral solution has been constructed for the water wave problem over a beach, that includes a logarithmic singularity at the shore line, and in the presence of a surface pressure excess. The logarithm, through its harmonic strength, provides a necessary source/sink term that balances the obstruction of the flow by the beach. Otherwise, the presence of such a singularity at the shoreline does not matter, because the waves do break before reaching this point and therefore a whole interval at this point lies outside the region of applicability of the linear theory. The method separates from the outset the singularity in the velocity potential and its derivatives at the origin, as well as the asymptotic behavior of the solution. It leads to formulae for the velocity potential satisfying the requirements of continuity to any degree of smoothness everywhere inside the domain of the flow. It also provides expressions for the strength of the logarithm, for the reflexion coefficient and for the coefficients of local perturbation in terms of unknown coefficients to be determined approximately by the satisfaction of the impermeability condition at the bottom. Cases with no reflexion are noted and the problem of an incoming wave against a cliff finds here an exact solution. The study deals with a variety of bottom shapes, and may be easily extended to cases involving an additional corrugation of the bottom over a finite interval, with submerged obstacles following the guidelines presented in^41^, or with different bottom boundary conditions. Unlike most of the existing literature, the present work contains numerical results for three beach topographies showing the systems of streamlines over the beach and the contiguous region with flat bottom. In all cases, the obtained values for the reflexion coefficient is nearly equal to unity, meaning that a system of standing waves establishes in the channel. This constraint, to our belief, is related to the special choice of the length parameter *a* involved in the finite Fourier transform.

The present results concerning the streamline distribution in the channel, and the possibility of wave trapping, may be of interest for environmental purposes, as it gives an indication about the way pollution propagates near the beaches.

### Supplementary Information

Supplementary Information.

## Data Availability

All data generated or analyzed during this study are included in this article.
