# Supplementary material for: Effect of a harmonic surface pressure on wave propagation over a beach
Source: Sci Rep. 2024 Jul 30;14:17547. doi: 10.1038/s41598-024-67443-6 (PMC11289401; doi:10.1038/s41598-024-67443-6)
Supplement: Supplementary file 1 — Supplementary Information. [file 41598_2024_67443_MOESM1_ESM.pdf]

Appendix A. Some coefficients

$$J_0 = \frac{1}{h}\int_{-h}^{0} \cosh^2 \lambda_0(\sigma+h)\, d\sigma = \frac{1}{2}\left(1+\frac{\sinh 2\lambda_0 h}{2\lambda_0 h}\right),$$
$$J_p = \frac{1}{h}\int_{-h}^{0} \cos^2 \lambda_p(\sigma+h)\, d\sigma = \frac{1}{2}\left(1+\frac{\sin 2\lambda_p h}{2\lambda_p h}\right), \qquad \lim_{p\to\infty} J_p = \frac{1}{2}.$$

$$\beta_0 = \frac{1}{J_0\lambda_0 h}\int_{-h}^{0}\left(\frac{a}{a^2+y^2}+\frac{a}{a^2+(y+2h)^2}\right)\cosh\lambda_0(y+h)\, dy,$$

$$\beta_p = \frac{1}{J_p\lambda_p h}\int_{-h}^{0}\left(\frac{a}{a^2+y^2}+\frac{a}{a^2+(y+2h)^2}\right)\cos\lambda_p(y+h)\, dy, \qquad \lim_{p\to\infty}\beta_p = 0.$$

$$I_{m0} = \frac{1}{h}\int_{-h}^{0}\cosh\frac{m\pi y}{a}\ \cosh\lambda_0(y+h)\, dy$$
$$= \begin{cases} \frac{1}{(\lambda_0 h)^2-\left(\frac{m\pi h}{a}\right)^2}\left(\lambda_0 h\sinh\lambda_0 h - \frac{m\pi h}{a}\sinh\frac{m\pi h}{a}\right), & m\neq 2N, \\ \frac{1}{2}\left(1+\frac{\kappa}{(\lambda_0 h)^2}\right)\cosh\lambda_0 h, & m=2N \end{cases}$$
$$J_{m0} = \frac{1}{h}\int_{-h}^{0}\sinh\frac{m\pi y}{a}\ \cosh\lambda_0(y+h)\, dy$$
$$= \begin{cases} \frac{\frac{m\pi h}{a}}{(\lambda_0 h)^2-\left(\frac{m\pi h}{a}\right)^2}\left(\cosh\frac{m\pi h}{a}-\cosh\lambda_0 h\right), & m\neq 2N, \\ -\frac{1}{2}\sinh\lambda_0 h, & m=2N \end{cases}$$
$$\lim_{m\to 0}\frac{J_{m0}}{\frac{m\pi h}{a}} = \frac{1-\cosh\lambda_0 h}{(\lambda_0 h)^2}$$

$$I_{mp} = \frac{1}{h}\int_{-h}^{0}\cosh\frac{m\pi}{a}y\ \cos\lambda_p(y+h)\, dy$$
$$= \frac{1}{(\lambda_p h)^2+\left(\frac{m\pi h}{a}\right)^2}\left(\lambda_p h\sin\lambda_p h+\frac{m\pi h}{a}\sinh\frac{m\pi h}{a}\right),$$
$$J_{mp} = \frac{1}{h}\int_{-h}^{0}\sinh\frac{m\pi}{a}y\ \cos\lambda_p(y+h)\, dy$$
$$= \frac{\frac{m\pi h}{a}}{(\lambda_p h)^2+\left(\frac{m\pi h}{a}\right)^2}\left(\cos\lambda_p h-\cosh\frac{m\pi h}{a}\right).$$
$$\lim_{m\to 0}\frac{J_{mp}}{\frac{m\pi h}{a}} = \frac{1}{(\lambda_p h)^2}\left(\cos\lambda_p h-1\right).$$

$$\rho_0 = \sum_{m=0}^{\infty}(-1)^m\,\frac{q_m^0}{m\pi}\left[W_m+\frac{h}{\kappa a}\left(\frac{\pi}{8}-Q_m\right)\right]\frac{J_{m0}}{J_0},$$
$$\rho_p = \sum_{m=0}^{\infty}(-1)^m\,\frac{q_m^0}{m\pi}\left[W_m+\frac{h}{\kappa a}\left(\frac{\pi}{8}-Q_m\right)\right]\frac{J_{mp}}{J_p}$$

$$
\begin{aligned}
G_m &= \frac{I_{m0}}{J_0} + \frac{a\kappa}{hm\pi}\frac{J_{m0}}{J_0}, \\
G_{mp} &= \frac{I_{mp}}{J_p} + \frac{a\kappa}{hm\pi}\frac{J_{mp}}{J_p}.
\end{aligned}
$$

$$
\begin{aligned}
c_1 &= \frac{i\nu}{\rho_0\kappa}\sum_{m=0}^{\infty}(-1)^m \frac{q_m^0}{m\pi}\frac{J_{m0}}{J_0}\,P_m, \qquad c_2 = \frac{1}{\rho_0\kappa}\left(I_{2N,0} + \frac{\kappa}{2N\pi}\frac{J_{2N,0}}{J_0}\right), \\
r_1 &= I_0 - i\beta_0 c_1, \qquad r_2 = -i\beta_0 c_2.
\end{aligned}
$$

$$
\begin{aligned}
s_p^{(1)} &= c_1\frac{\rho_p\kappa - \beta_p\coth\lambda_p a}{1 + e^{-\lambda_p a}\coth\lambda_p a} + \frac{i\nu}{1 + e^{-\lambda_p a}\coth\lambda_p a}\sum_{m=0}^{\infty}(-1)^m \frac{q_m^0}{m\pi}\,P_m\,\frac{J_{mp}}{J_p}, \\
s_p^{(2)} &= c_2\frac{\rho_p\kappa - \beta_p\coth\lambda_p a}{1 + e^{-\lambda_p a}\coth\lambda_p a} + \frac{1}{1 + e^{-\lambda_p a}\coth\lambda_p a}\left(I_{2N,p} + \frac{\kappa}{2N\pi}\frac{J_{2N,p}}{J_p}\right)
\end{aligned}
$$

Appendix B. Components of the velocity potential and streamline functions

$$
\begin{aligned}
\Phi_0^{(0)}(x,y) &= (I_0+r_1)\cosh\lambda_0(y+h)\cos\lambda_0 x \\
&+ i\sum_{m=0}^{K}\frac{q_m^0}{m\pi}\left(\nu P_m - ic_1\kappa\left[W_m+\frac{h}{\kappa a}\left(\frac{\pi}{8}-Q_m\right)\right]\right)\sinh\frac{m\pi y}{a}\cos\frac{m\pi x}{a} \\
&- \sum_{p=1}^{P}\left(s_p^{(1)}e^{-\lambda_p a}+c_1\beta_p\right)\frac{\cosh\lambda_p x}{\sinh\lambda_p a}\cos\lambda_p(y+h)+c_1 Z(x,y)
\end{aligned}
$$

$$
\begin{aligned}
\Phi_m^{(1)}(x,y) &= \left(\frac{\cosh\frac{m\pi y}{a}}{\cosh\frac{m\pi h}{a}}+\frac{a}{h}\frac{1}{m\pi}\,\kappa\,\frac{\sinh\frac{m\pi y}{a}}{\cosh\frac{m\pi h}{a}}\right)\cos\frac{m\pi x}{a} \\
&- \frac{i\beta_0}{\kappa\rho_0}(-1)^m\frac{G_m}{\cosh\frac{m\pi h}{a}}\cosh\lambda_0(y+h)\cos\lambda_0 x \\
&+ (-1)^m\frac{G_m}{\cosh\frac{m\pi h}{a}}W(x,y) \\
&- (-1)^m\sum_{p=1}^{P}\left[\frac{e^{-\lambda_p a}}{1+e^{-\lambda_p a}\coth\lambda_p a}\left(\frac{\kappa\rho_p-\beta_p\coth\lambda_p a}{\kappa\rho_0}\frac{G_m}{\cosh\frac{m\pi h}{a}}+\frac{G_{mp}}{\cosh\frac{m\pi h}{a}}\right)\right. \\
&\left.- \frac{\beta_p}{\kappa\rho_0}\frac{G_m}{\cosh\frac{m\pi h}{a}}\right]\frac{\cosh\lambda_p x}{\sinh\lambda_p a}\cos\lambda_p(y+h)+\frac{1}{\kappa\rho_0}(-1)^m\frac{G_m}{\cosh\frac{m\pi h}{a}}Z(x,y),
\end{aligned}
$$

$$
\Phi_0^{(2)}(x,y) = i\cosh\lambda_0(y+h)\sin\lambda_0 x+\cosh\lambda_0(y+h)\cos\lambda_0 x
$$

$$
\begin{aligned}
\Phi_p^{(2)}(x,y) &= -\coth\lambda_p a\cosh\lambda_p(a-x)\cos\lambda_p(y+h) \\
&+ \frac{\coth\lambda_p a\left(\coth\lambda_p a-1\right)}{1+e^{-\lambda_p a}\coth\lambda_p a}\cosh\lambda_p x\cos\lambda_p(y+h), \qquad p=1,2,3,\cdots,
\end{aligned}
$$

$$
\begin{aligned}
\Phi^{(3)}(x,y) &= \cosh\frac{2N\pi y}{a}\cos\frac{2N\pi x}{a}+\frac{\kappa}{2N\pi}\sinh\frac{2N\pi y}{a}\cos\frac{2N\pi x}{a} \\
&+ r_2\cosh\lambda_0(y+h)\cos\lambda_0 x \\
&- \sum_{p=1}^{P}\left(s_p^{(2)}e^{-\lambda_p a}+c_2\beta_p\right)\frac{\cosh\lambda_p x}{\sinh\lambda_p a}\cos\lambda_p(y+h)+c_2 Z(x,y),
\end{aligned}
$$

where

$$
W(x,y)=\lim_{R\to\infty}\frac{\displaystyle\sum_{r=1}^{R}(-1)^r\frac{q_r^0}{r\pi}\left[W_r+\frac{h}{\kappa a}\left(\frac{\pi}{8}-Q_r\right)\right]\frac{\sinh\frac{r\pi y}{a}}{G_R}\cos\frac{r\pi x}{a}}{\frac{\rho_0}{G_R}}.
$$

The corresponding stream functions are:

$$\begin{aligned}
\Psi_0^{(0)}(x,y) =& -(I_0+r_1)\sinh\lambda_0(y+h)\sin\lambda_0 x \\
& - i\sum_{m=0}^{K}\frac{q_m^0}{m\pi}\left(\nu P_m - ic_1\kappa\left[W_m+\frac{h}{\kappa a}\left(\frac{\pi}{8}-Q_m\right)\right]\right)\cosh\frac{m\pi y}{a}\sin\frac{m\pi x}{a} \\
& - \sum_{p=1}^{P}\left(s_p^{(1)}e^{-\lambda_p a}+c_1\beta_p\right)\frac{\sinh\lambda_p x}{\sinh\lambda_p a}\sin\lambda_p(y+h)+c_1 Z^c(x,y)
\end{aligned}$$

$$\begin{aligned}
\Psi_m^{(1)}(x,y) =& -\left(\frac{\sinh\frac{m\pi y}{a}}{\cosh\frac{m\pi h}{a}}+\frac{a}{h}\frac{1}{m\pi}\kappa\frac{\cosh\frac{m\pi y}{a}}{\cosh\frac{m\pi h}{a}}\right)\sin\frac{m\pi x}{a} \\
& + \frac{i\beta_0}{\kappa\rho_0}(-1)^m\frac{G_m}{\cosh\frac{m\pi h}{a}}\sinh\lambda_0(y+h)\sin\lambda_0 x \\
& + (-1)^m\frac{G_m}{\cosh\frac{m\pi h}{a}}W^c(x,y) \\
& - (-1)^m\sum_{p=1}^{P}\left[\frac{e^{-\lambda_p a}}{1+e^{-\lambda_p a}\coth\lambda_p a}\left(\frac{\kappa\rho_p-\beta_p\coth\lambda_p a}{\kappa\rho_0}\frac{G_m}{\cosh\frac{m\pi h}{a}}+\frac{G_{mp}}{\cosh\frac{m\pi h}{a}}\right)\right. \\
& \left. - \frac{\beta_p}{\kappa\rho_0}\frac{G_m}{\cosh\frac{m\pi h}{a}}\right]\frac{\sinh\lambda_p x}{\sinh\lambda_p a}\sin\lambda_p(y+h)+\frac{1}{\kappa\rho_0}(-1)^m\frac{G_m}{\cosh\frac{m\pi h}{a}}Z^c(x,y),
\end{aligned}$$

$$\Psi_0^{(2)}(x,y) = i\sinh\lambda_0(y+h)\cos\lambda_0 x-\sinh\lambda_0(y+h)\sin\lambda_0 x$$

$$\begin{aligned}
\Psi_p^{(2)}(x,y) =& \coth\lambda_p a\sinh\lambda_p(a-x)\sin\lambda_p(y+h) \\
& + \frac{\coth\lambda_p a\,(\coth\lambda_p a-1)}{1+e^{-\lambda_p a}\coth\lambda_p a}\sinh\lambda_p x\sin\lambda_p(y+h), \qquad p=1,2,3,\cdots,
\end{aligned}$$

$$\begin{aligned}
\Psi^{(3)}(x,y) =& -\sinh\frac{2N\pi y}{a}\sin\frac{2N\pi x}{a}-\frac{\kappa}{2N\pi}\cosh\frac{2N\pi y}{a}\sin\frac{2N\pi x}{a} \\
& - r_2\sinh\lambda_0(y+h)\sin\lambda_0 x \\
& - \sum_{p=1}^{P}\left(s_p^{(2)}e^{-\lambda_p a}+c_2\beta_p\right)\frac{\sinh\lambda_p x}{\sinh\lambda_p a}\sin\lambda_p(y+h)+c_2\,Z^c(x,y),
\end{aligned}$$

with

$$W^c(x,y) = -\lim_{R\to\infty}\frac{\displaystyle\sum_{r=1}^{R}(-1)^r\frac{q_r^0}{r\pi}\left[W_r+\frac{h}{\kappa a}\left(\frac{\pi}{8}-Q_r\right)\right]\frac{\cosh\frac{r\pi y}{a}}{G_R}\sin\frac{r\pi x}{a}}{\frac{\rho_0}{G_R}}.$$
